# Regulation of Sister Chromosome Cohesion by the Replication Fork Tracking Protein SeqA

**DOI:** 10.1371/journal.pgen.1003673

**Published:** 2013-08-22

**Authors:** Mohan C. Joshi, David Magnan, Timothy P. Montminy, Mark Lies, Nicholas Stepankiw, David Bates

**Affiliations:** 1 Department of Molecular and Human Genetics, Baylor College of Medicine, Houston, Texas, United States of America; 2 Interdepartmental Program in Cell and Molecular Biology, Baylor College of Medicine, Houston, Texas, United States of America; Institute of Molecular and Cell Biology (IMCB), A*STAR, Singapore

## Abstract

Analogously to chromosome cohesion in eukaryotes, newly replicated DNA in *E. coli* is held together by inter-sister linkages before partitioning into daughter nucleoids. In both cases, initial joining is apparently mediated by DNA catenation, in which replication-induced positive supercoils diffuse behind the fork, causing newly replicated duplexes to twist around each other. Type-II topoisomerase-catalyzed sister separation is delayed by the well-characterized cohesin complex in eukaryotes, but cohesion control in *E. coli* is not currently understood. We report that the abundant fork tracking protein SeqA is a strong positive regulator of cohesion, and is responsible for markedly prolonged cohesion observed at “snap” loci. Epistasis analysis suggests that SeqA stabilizes cohesion by antagonizing Topo IV-mediated sister resolution, and possibly also by a direct bridging mechanism. We show that variable cohesion observed along the *E. coli* chromosome is caused by differential SeqA binding, with *oriC* and snap loci binding disproportionally more SeqA. We propose that SeqA binding results in loose inter-duplex junctions that are resistant to Topo IV cleavage. Lastly, reducing cohesion by genetic manipulation of Topo IV or SeqA resulted in dramatically slowed sister locus separation and poor nucleoid partitioning, indicating that cohesion has a prominent role in chromosome segregation.

## Author Summary

Sister chromosome cohesion in eukaryotes maintains genome stability by mediating chromosome segregation and homologous recombination-dependent DNA repair. Here we have investigated the mechanism of cohesion regulation in *E. coli* by measuring cohesion timing in a broad set of candidate mutant strains. Using a sensitive DNA replication and segregation assay, we show that cohesion is controlled by the conserved DNA decatenation enzyme Topo IV and the abundant DNA binding protein SeqA. Results suggest that cohesion occurs in *E. coli* by twisting of replicated duplexes around each other behind the replication fork, and immediate resolution of cohered regions is blocked by SeqA. SeqA binds to a sliding 300–400 kb window of hemimethylated DNA behind the fork, and regions binding more SeqA experience longer cohesion periods. An analogous decatenation inhibition function is carried out by the cohesin complex in eukaryotes, indicating that cells mediate pairing and separation of replicated DNA by a conserved mechanism. In both cases, mismanaged cohesion results in failed or inefficient chromosome segregation.

## Introduction

Chromosome dynamics studies in *E. coli* using either fluorescent *in situ* hybridization (FISH) or fluorescent repressor proteins bound to arrays of operator sequences (FROS) have shown that there is a significant time delay between passage of the replication fork and separation of replicated sequences into two visible foci [1]–[6]. Comprehensive surveys across the *E. coli* chromosome indicate that this delay is ∼10 minutes at most sites [3], [6], suggesting that a several hundred kilobase sliding window of sister “non-separation” (i.e., cohesion) follows each replication fork. Superimposed on this brief and progressive cohesion program, three regions have been identified that exhibit much longer cohesion, including the replication origin, *oriC* and two broad domains on the right chromosome arm [3], [6]. The two late-splitting right arm regions, which we term “snaps”, are further unique in that their cohesion is lost simultaneously and is accompanied by a major global nucleoid reorganization event that gives rise to a bilobed nucleoid morphology [6]. This abrupt transition involves significant nucleoid expansion [7] and comprises a sister individualization step in which each nucleoid lobe contains one partially replicated daughter chromosome [6]. These data led us to propose that snap regions promote efficient chromosome segregation by resisting global sister chromosome separation until an appropriate time in the cell cycle. In this light, snaps may be analogous to eukaryotic centromere elements, which provide essential tension for microtubule-assisted chromosome segregation (Discussion).

Although there is no known bacterial equivalent of the eukaryotic cohesin complex that holds sisters together by a covalent ring structure [8], several lines of evidence suggest that colocalized sister regions in *E. coli* form a molecular complex. First, for the duration of the segregation delay, “cohered” regions remain within the resolution limit of fluorescence microscopy, ∼230 nm [6]. Subsequent separation is very rapid (1–2 µm in 1–3 min; [9]), implying that segregation tension is counteracted by covalent linkages during cohesion. Second, disruption of the *oriC* partitioning apparatus by eliminating MukB does not cause increased *oriC* cohesion [10], as would be expected if newly replicated regions merely passively colocalized until acted upon by segregation machinery. Third, a critical component of cohesion in *E. coli* appears to be the decatenating enzyme topoisomerase IV (Topo IV), suggesting that part or all of the basis for cohesion is entanglement of replicated DNA behind the fork [4]. Fourth, inter-sister recombination exchanges occur more frequently between cohered loci [11], indicating that homologous sequences physically interact during the colocalization period, and are not merely in the same subcellular vicinity.

Currently, the only known mediator of cohesion in *E. coli* is the well-conserved type-II topoisomerase, Topo IV. Inactivation of Topo IV via a temperature-sensitive mutation led to a reduction in sister separation near *oriC* [4], [11], and also within the terminus region [11], implying that Topo IV modulates cohesion across the *E. coli* chromosome. Topo IV, which relaxes positively supercoiled DNA molecules by a double-stranded cut/passage/ligation mechanism [12], was initially thought to act primarily in the terminus region, where converging replication forks generate maximal positive supercoiling. However, Topo IV is also present at the replisome continually during replication [13], [14], which suggests that positive supercoils frequently migrate behind the replication fork, causing nascent sister duplexes to wind around each other in a precatenane structure. Single molecule studies estimate that Topo IV, present at ∼1000 molecules per cell, has a total unlinking capacity of ∼6000 strand passages per second [15], several orders of magnitude faster than the rate at which precatenanes are predicted to form [16]. In contrast, cohesion lasts at minimum ∼7 minutes and up to 30 minutes along snap regions [6]. Thus, it appears that either cohesion involves another molecular linking component besides precatenanes, or, that Topo IV is negatively regulated by an unknown factor.

To investigate how sister cohesion is regulated in *E. coli*, we analyzed cohesion timing in a broad range of chromosome structure and segregation mutants. Candidate cohesion regulatory proteins included the SMC-like proteins MukB and RecN, the nucleoid associated proteins HU, IHF and Fis, the replication fork tracking protein SeqA and its binding regulator Dam, and Topo IV. MukB and RecN are the only *E. coli* proteins with structural similarity to eukaryotic cohesin [17], and could potentially promote cohesion by forming protein bridges across sister chromosomes [1]. The “histone-like” proteins HU, IHF and Fis, and the abundant DNA binding protein SeqA, are important for maintaining nucleoid structure and supercoiling [18], and could also modulate cohesion through bridging or by net effects on chromosome compaction [19]. SeqA in particular is well positioned to regulate cohesion because it binds strongly and specifically to newly replicated DNA [20]. As DNA exits the replication fork the newly synthesized strand is unmethylated for a period of 5–10 minutes, before remethylation by Dam methylase [21]. During this period of hemimethylation, GATC sequences are bound by SeqA, with potentially several hundred molecules bound behind each fork [19], and SeqA-GFP fusions forming large foci near or adjacent to sites of DNA replication [19], [22], [23]. It may not be coincidence that hemimethylation and cohesion periods (of typical non-snap loci) are very near equal. Importantly, in addition to a direct (bridging) mechanism, any of these proteins could regulate cohesion indirectly by affecting the processing of DNA catenanes. Supporting this idea, both SeqA and MukB interact with Topo IV *in vivo* and have been shown to strongly affect Topo IV decatenase activity *in vitro* [24]–[26].

## Results

### Chromosome cohesion is oppositely regulated by SeqA and Topo IV

**Figure 1:**
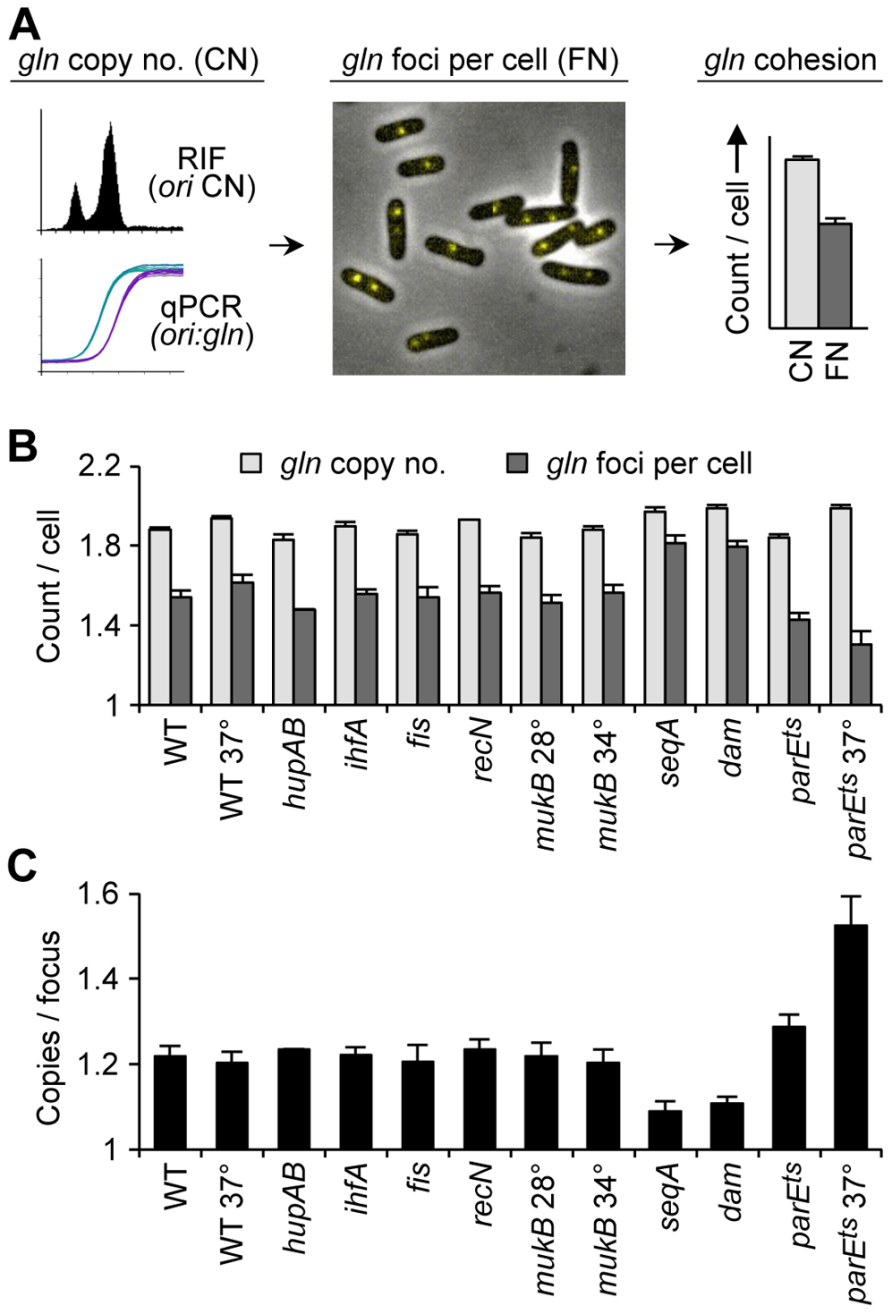
Cohesion in *E. coli* chromosome structure mutants. (A) Cohesion assay. Cohesion values (*gln* copies per focus) are determined by independently measuring *gln* copy number and resolvable *gln* foci per cell in asynchronous exponential cells bearing a *tetO* array at *gln* and expressing fluorescent TetR-YFP. See text for details. (B) *gln* copy number and TetR-YFP foci per cell in wild-type and mutant cells bearing a *tetO* array at the *gln* locus. Cells were grown exponentially in minimal alanine media, and *gln* copy number and foci per cell were determined (Materials and Methods). Cells were grown continually at 30°C or shifted to the indicated temperature 4 hours prior to analysis. Values are means of three independent experiments ±1 standard deviation (SD). (C) Cohesion values (average number of *gln* copies per *gln* focus) for candidate mutant strains. Focus values were adjusted for small inefficiencies of fluorescent detection (Materials and Methods).

To identify factors involved in the regulation of chromosome cohesion, we performed a candidate screen for mutants that have increased or decreased sister cohesion. Mutants were selected that displayed both a moderate to severe chromosome segregation phenotype and abnormal nucleoid shape or compaction (Introduction). Cohesion in these strains was determined in exponential cultures at the well-characterized *gln* locus using our standard non-synchronized cell assay (Figure 1A). *gln* copy number is determined by first measuring *oriC* copies per cell by rifampicin runoff flow cytometry, then measuring the relative ratio of *gln* copies to *oriC* copies by quantitative real time PCR (qPCR). In parallel, *gln* foci per cell is determined by FROS in which a tandem array of *tetO* binding sequences is inserted into the chromosome and subsequently bound by a fluorescent TetR-YFP fusion protein. The duration of sister co-localization (cohesion) is then proportional to the difference in *gln* copy number and *gln* foci per cell. This assay requires high efficiency of fluorescence detection (below) and sufficient resolution of segregated loci. Because initial segregation velocities are rapid, about 0.4 µm/min in the current study with final positions 3–10 times greater than the resolution distance of light microscopy (shown below), we estimate that sister loci appear as two fluorescent foci <30 seconds after loss of sister cohesion. Although this cohesion assay is valid under any growth rate, cells were grown in minimal media supplemented with alanine or succinate as indicated to minimize overlapping replication cycles, which simplified microscopy analysis.

The *gln* locus, located on the right chromosome arm 130 kb from *oriC*, normally exhibits a 30 minute cohesion period under similar growth conditions [2], [6]. In the present study, *gln* was present at 1.9 copies per cell in wild-type cells, indicating that most cells had one or two chromosomes and that replication initiation occurred relatively early in the cell cycle (Figure 1B; Figure S1). Mutant strains had similar copy numbers, ranging between 1.8 and 2.0 *gln* loci per cell. Average *gln* TetR-YFP foci per cell for *wildtype*, *hupAB*, *ihfA*, *fis*, *recN* and *mukB*, was ∼20% lower than the respective *gln* copy number (Figure 1B). This suggests that a significant fraction of foci in these strains harbored two colocalized *gln* loci (separated by <0.2 µm) in a state of cohesion. Due to the inherent limitations of fluorescence labeling and imaging, the observed number of fluorescent *tetO*/TetR-YFP complexes per cell is slightly undervalued, leading to an overestimation of cohesion. To correct for this, efficiencies were determined for each FROS experiment (94%±4%), and raw focus counts were adjusted (≤+0.10 foci per cell; Materials and Methods). This method was verified by determining the number of *gln* foci in a population of non-replicating, and presumably cohesion-less, stationary phase cells (Figure S2). Resulting *gln* copies per focus values for *wildtype* and most mutants were 1.22±0.03 (Figure 1C), indicating that most strains, including Δ*mukBEF*, had normal *gln* cohesion (∼30 min; [6]).

In contrast to WT, Δ*seqA* cells contained only 1.09 *gln* copies per focus (Figure 1C), indicating that *gln* cohesion is reduced ∼60% in the absence of SeqA protein. A *dam* mutant, which is defective in GATC methylation and thus does not target SeqA to newly replicated DNA, had nearly identical *gln* cohesion as *ΔseqA* as expected. An opposite effect on cohesion was seen in cells with reduced levels of Topo IV. Cells bearing a *parE10*(Ts) mutation that produces a defective Topo IV protein at 42°C [27], [28] showed a sharp increase in cohesion when incubated at the semi-permissive temperature of 37°C (Figure 1C), indicating that Topo IV mediates cohesion at an arm locus in addition to its role at *oriC* and *ter* [4], [11].

### Synchronized cell analysis

**Figure 2:**
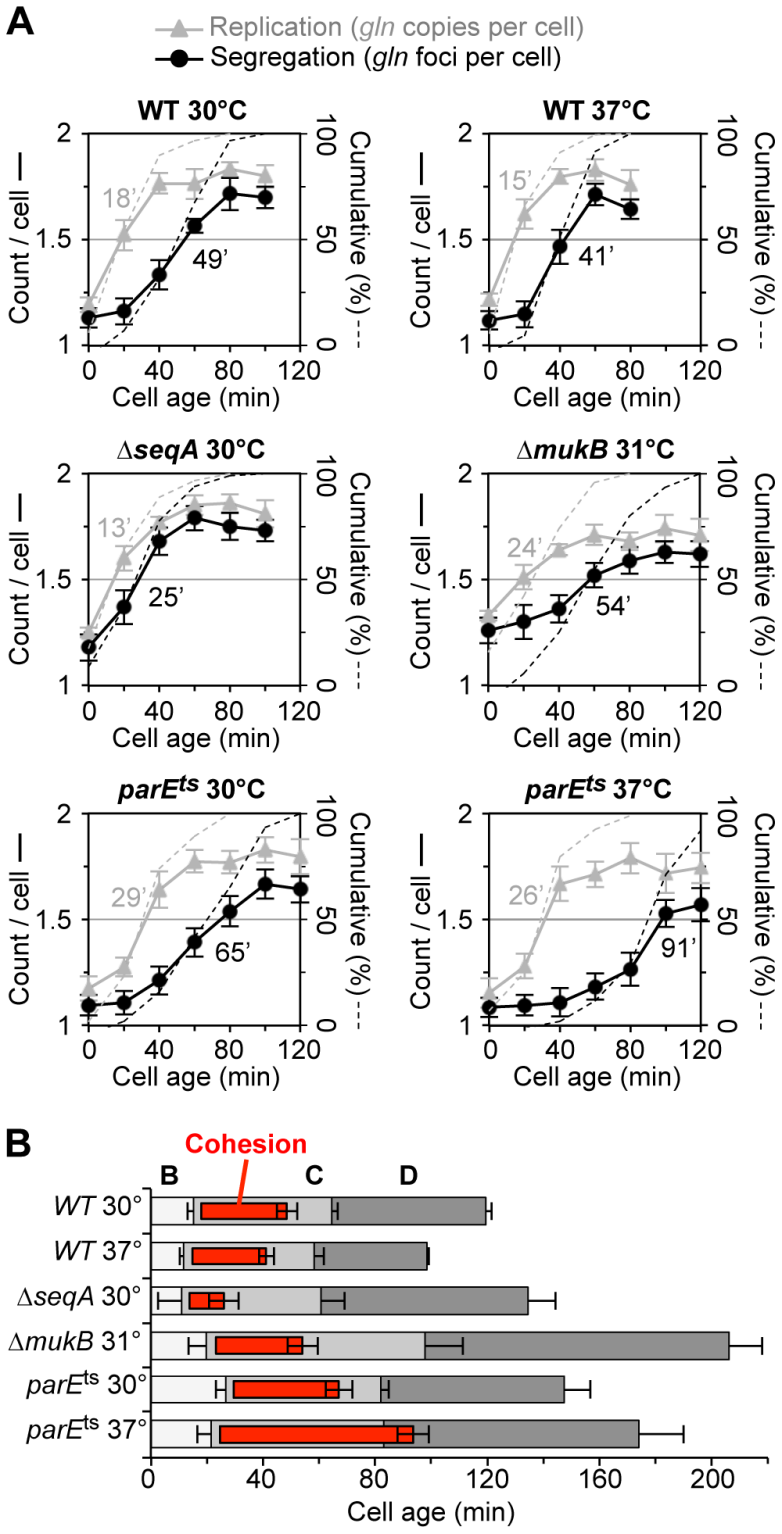
Synchronized cell analysis of Δ*seqA*, Δ*mukB and parE10* strains. (A) Timing of replication and segregation of the *gln* locus. Cells were synchronized by baby machine in minimal alanine media at the indicated temperature and assayed for *gln* copy number (grey triangles) and number of TetR-YFP foci per cell (black circles) during the cell cycle. Values are means of two experiments ±1 SD. Dashed lines indicate the cumulative percentage of cells in the synchronized fraction that have replicated and segregated the *gln* locus (right ordinate), with times at 50% shown in minutes after birth. (B) Cell cycle diagrams are shown based on the timing of *oriC*, *gln* and *ter* duplication by qPCR (Materials and Methods). Doubling times in minimal alanine media were: WT/30°C, 119 min; WT/37°C, 98 min; Δ*seqA*/30°C, 134 min; Δ*mukB*/30°C, 211 min; *parE10*/30°C, 147 min; *parE10*/37°C, 174 min.

Although we infer from non-synchronized “batch” culture analysis that Δ*seqA* and *parE10* cells have shorter and longer *gln* cohesion periods respectively (Figure 1), it is possible that these mutants produce mixed populations of cells (with altered replication timing) that could bias cohesion measurements. To address this, we examined the dynamics of *gln* cohesion during the cell cycle by synchronized cell analysis (Figure 2). Wild-type, Δ*seqA*, Δ*mukBEF*, and *parE10* mutant cells were synchronized by the baby machine method, which results in 75–85% synchrony and cells that are unperturbed for rates of mass increase, DNA replication and cell division [29]. Synchronized cells were then assayed for *gln* replication and *gln* splitting for two hours after cell birth, the equivalent of one cell cycle for wild-type cells at 30°C. In wild-type cells at both 30°C and 37°C, *gln* copy numbers rose steeply after cell birth (Figure 2A, top panels, gray), followed by an increase in *gln* foci per cell ∼30 minutes later (black). Integrating the area under the raw data curves at each time point yields cumulative curves (Figure 2A, dashed lines), which describe the percentage of cells among the synchronous fraction that have replicated or segregated over time [2]. Cohesion periods are thus defined as the time interval between the replication and segregation cumulative curves. For wild-type cells, *gln* cohesion lasted 31 minutes at 30°C and 26 minutes at 37°C in agreement with previous studies [2], [6].

As was seen with *wildtype*, *ΔseqA* cells exhibited steep increases in *gln* copies and *gln* foci during the cell cycle. However, *gln* foci split much sooner after replication in Δ*seqA* cells (Figure 2A, middle left panel). Cumulative curve analysis indicates that cohesion lasted about 12 minutes in Δ*seqA* cells, about 1/3 the normal duration of *gln* cohesion (Figure 2B). Confirming results from our initial screen, *mukB* mutant cells exhibited ∼30 minutes of *gln* cohesion at 31°C, the highest temperature that supported steady state growth (Figure 2A, middle right panel). Synchrony in *mukB* cells was relatively poor (note shallow curves for *gln* copy number and foci per cell), but *gln* splitting was clearly delayed after replication to a similar extent as *wildtype*. We conclude that like *oriC* [10], *gln* cohesion does not require MukB protein. In contrast, *parE10* cells showed severely delayed *gln* splitting at the semi-permissive temperature of 37°C (Figure 2A, lower right panel). Under this condition, *gln* cohesion lasted about 65 minutes, 2-fold longer than when cells were grown at 30°C (left panel). Importantly, the segregation delay was not caused by indirect effects of temperature, as wild-type cells showed an even shorter cohesion period at 37°C compared to 30°C. Interestingly, both *seqA* and *parE10* cells had significantly longer post-replication D periods than wild-type cells at the same temperature (Figure 2B), indicating that cell division was delayed. This delay may stem from late sister segregation caused by improper cohesion, although indirect effects on segregation cannot be ruled out (*mukB* cells also had extended D periods, Figure 2B).

### SeqA binding determines the duration of cohesion

**Figure 3:**
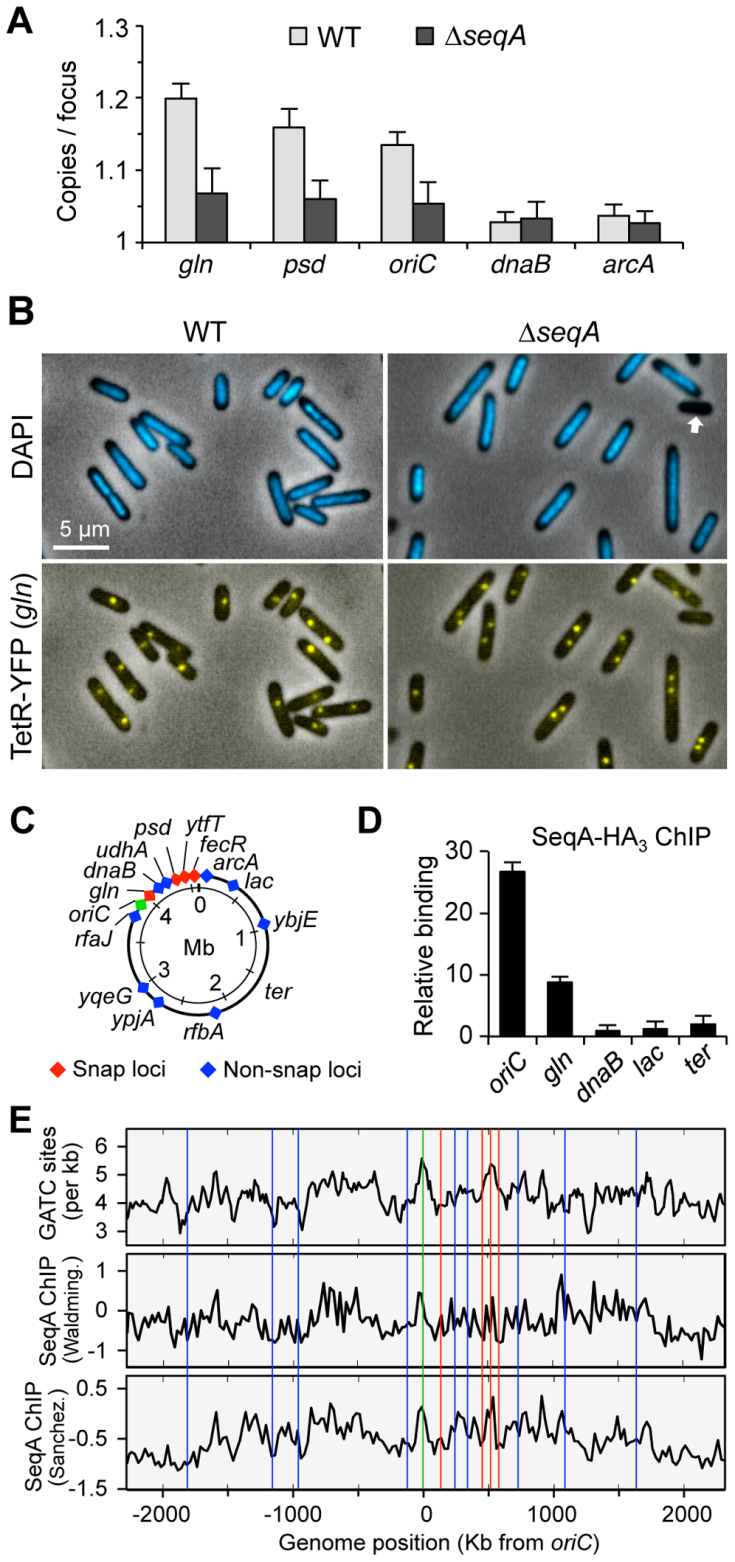
SeqA is responsible for delayed separation at late-splitting loci. (A) Loss of SeqA reduces cohesion at snaps and *oriC*, but not at non-snap loci *dnaB* and *arcA*. WT and *ΔseqA* strains bearing *tetO* arrays at each of the five loci shown were grown in minimal alanine media and assayed for cohesion as described in Figure 1 (3 independent experiments ±1 SD). (B) Representative micrographs of *wildtype* and Δ*seqA* cells showing nucleoids (DAPI) and *gln* TetR-YFP foci. Δ*seqA* cells exhibit ∼2% anucleate cells (arrow). (C) *E. coli* chromosome map with cohesion-characterized loci [6]. Snap loci (red) and *oriC* (green) have prolonged cohesion periods (19–30 min); non-snap loci (blue) have short cohesion periods (7–10 min). (D) Sites with prolonged cohesion bind more SeqA. SeqA binding levels at 5 loci shown were determined by ChIP-qPCR. Relative binding (2^−ΔΔCt^) indicates SeqA binding relative to the poorest binding sequence, *dnaB*. (E) Genomic analysis of GATC frequency, SeqA binding, and cohesion. GATC per kb (top panel), and SeqA binding from two SeqA ChIP-chip studies; Waldminghaus et. al. [31] (middle panel) and Sanchez-Romero et. al. [32] (lower panel), 40-kb moving average of SeqA binding is shown (log_2_ ratio of IP to input fluorescence). Positions of cohesion-characterized loci are shown as colored vertical lines.

Synchronized cell analysis showed that *gln* cohesion, normally lasting ∼30 minutes, was reduced to ∼12 minutes in the absence of SeqA protein. To test whether SeqA mediates cohesion at sites other than *gln*, cohesion was measured in wild-type and Δ*seqA* cells at 5 chromosomal loci (see map, Figure 3C): two late-splitting snap loci (*gln* and *psd*), two fast-splitting non-snap loci (*dnaB* and *arcA*), and *oriC*, which exhibits late-splitting but with much different timing than snap loci [6]. At *oriC* and both snap sites (*gln* and *psd*), cohesion was ∼60% reduced in Δ*seqA* compared to *wildtype* (Figure 3A). Δ*seqA* cells were slightly elongated (3.1 µm compared to 2.4 µm for WT) with ∼2% anucleate cells (Figure 3B, arrow), suggesting that nucleoid segregation is partially defective. Although it is possible that aberrant replication initiation causes these effects, under the current slow growth conditions the Δ*seqA* initiation phenotype is greatly suppressed as indicated by only a 5-minute advanced initiation timing (Figure 2), suggesting that segregation problems are due to reduced cohesion (Discussion). Unlike snap cohesion, cohesion at the two non-snap loci *dnaB* and *arcA* was not measurably different in *ΔseqA* cells (Figure 3A), although any subtle (<20%) changes in cohesion at these sites might be below our current level of detection. Subsequent experiments showing that an overabundance of SeqA causes prolonged cohesion at *dnaB* suggest that SeqA is able to promote cohesion at non-snap loci under some conditions (below).

To evaluate whether sites exhibiting higher cohesion are enriched in SeqA binding, we performed chromatin immunoprecipitation against HA-tagged SeqA protein, followed by site-specific analysis of immunoprecipitated DNA by qPCR (ChIP-qPCR). As expected, *oriC* DNA bound much more SeqA than the non-snap locus *dnaB* (25-fold enrichment, Figure 3D). The snap locus *gln* also showed elevated SeqA binding (10-fold over *dnaB*), whereas two other non-snap loci, *lac* and *ter*, exhibited similar low levels of SeqA binding. The relatively high abundance of *oriC* DNA on the SeqA complexes, probably reflects a small but very dense cluster of GATC sequences within the origin itself (known as the 13-mers, see [30]).

To examine whether the above correlation between cohesion and SeqA binding extend to other sites on the chromosome, we compared cohesion at 15 characterized loci from our earlier study [6] to genomic SeqA binding data from two *E. coli* microarray ChIP-chip studies [31], [32] as well as the frequency of GATC sequences (Figure 3E). Several insights emerge from this analysis. First, large-scale SeqA binding trends (40-kb moving average shown) from both ChIP-chip studies are quite similar, and generally reflect the density of GATC sequences, but not perfectly. This likely reflects the fact that cooperative SeqA binding is optimal when adjacent GATC spacing places them on the same helical face [19], thus some GATC sequences do not bind SeqA well. Second, several prominent peaks and valleys are present in the SeqA binding plots, and these fluctuations correspond generally to locations of snaps and non-snaps, respectively. Third, a higher resolution analysis of the ChIP-chip data near our sites of interest (5-kb moving average, Figure S3) resulted in an improved correlation between SeqA binding and cohesion, suggesting that cohesion levels may be regulated by local variations in SeqA binding (Discussion).

### Cohesion at snap and non-snap loci after Topo IV inactivation

**Figure 4:**
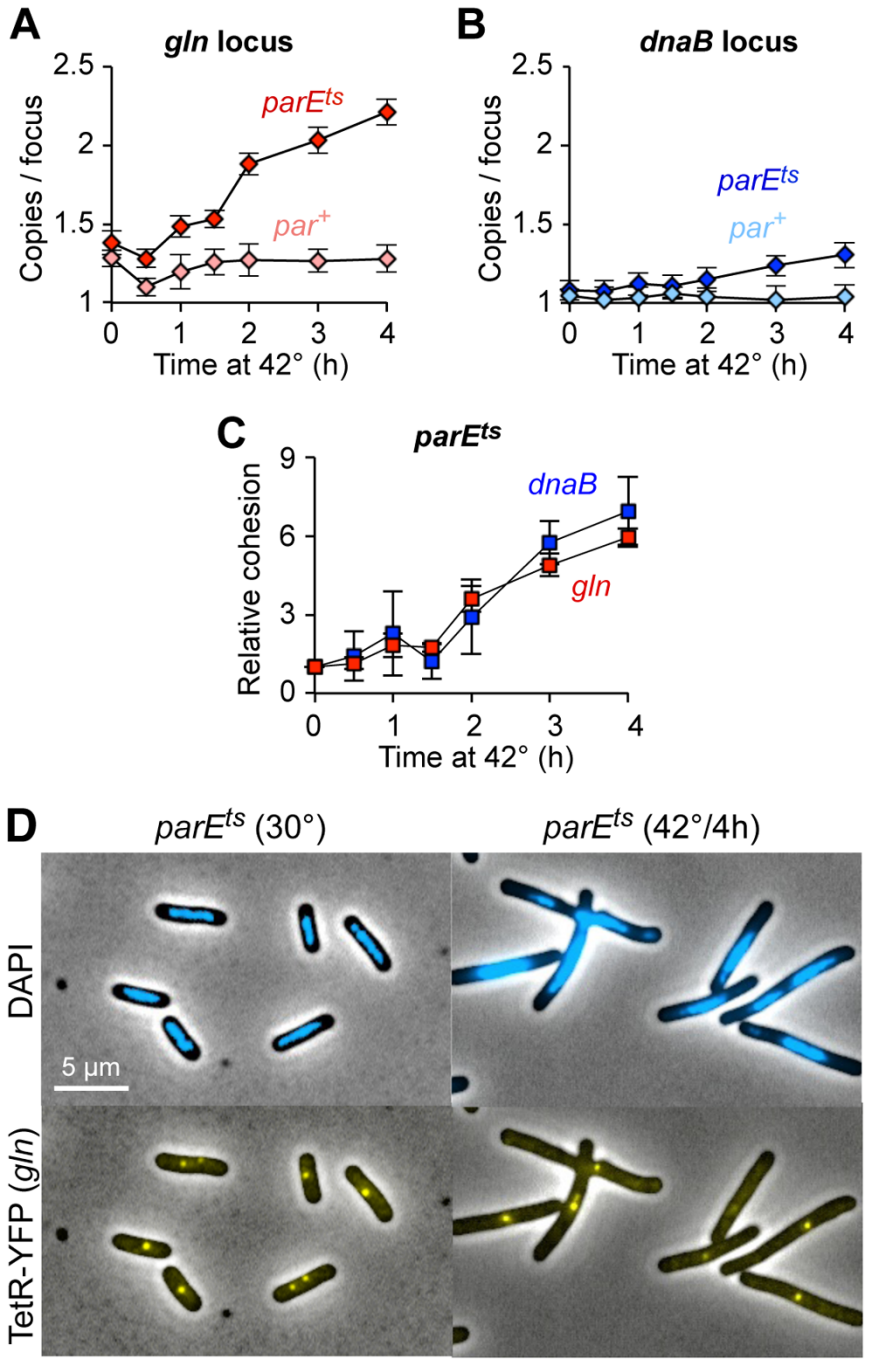
Topo IV reduces cohesion at both snap and non-snap loci. (A–B) Raw cohesion values at *gln* (*A*) and *dnaB* (*B*) after Topo IV inactivation. Copy number per TetR-YFP focus was determined in *parE10* (dark shaded symbols) and *par^+^* control (light shaded symbols) cells after shift to restrictive temperature. Values are means of 3–4 experiments ±1 SD. Cells were grown to early log phase at 30°C in minimal succinate medium, shifted to 42°C, and assayed as described in Figure 1. (C) Relative *gln* and *dnaB* cohesion after Topo IV inactivation, normalized to 30°C. The difference in cohesion (copies/focus) between *parE10* and WT at each time point relative to the difference at t = 0 is shown for *gln* and *dnaB*. (D) Representative micrographs of wild-type and *parE10* cells at 30°C and 42°C.

To further evaluate the role of Topo IV in regulating cohesion, we measured copy number and foci per cell at a snap locus (*gln*) and a non-snap locus (*dnaB*) after inactivation of Topo IV via a temperature sensitive mutation (Figure 4). WT or *parE10* cells were grown at 30°C in minimal succinate media to early log phase (WT doubling time ∼90 min), shifted to the non-permissive temperature of 42°C, and assayed as described in Figure 1. Cohesion at the *gln* locus increased steadily in *parE10* cells after temperature upshift, reaching a maximum of ∼2.2 copies per focus by 4 hours (Figure 4A, dark red symbols). Cohesion at the fast-splitting non-snap *dnaB* locus was also prolonged by depletion of Topo IV (Figure 4B), reaching a maximum of ∼1.4 copies per focus by 4 hours (dark blue symbols). Cohesion did not significantly change at either locus in *par^+^* cells after temperature upshift (Figure 4AB, light shaded symbols). Interestingly, in all four cases cohesion decreased during the first 30 minutes after temperature upshift, suggesting that high temperature induced conformational changes to DNA that facilitated cohesion loss. Increased cohesion in *parE10* cells was not due to replication fork stalling, as shown by complete replication runoff after rifampicin treatment (Figure S4A) and continued DNA synthesis by radioactive thymidine incorporation (Figure S4B).

Although absolute cohesion levels were higher at *gln* than at *dnaB* under all conditions, the relative rate of increase in cohesion after Topo IV inactivation was similar for both loci (Figure 4C). Thus, *gln* and *dnaB* were equally sensitive to a lack of Topo IV, further implying that all sequences experience similar levels of catenation. We postulate that higher observed cohesion at *gln* and other snap loci is caused by another mechanism at these sites, presumably mediated by SeqA, which either inhibits Topo IV and/or directly facilitates sister cohesion (Discussion). Additionally, the present data provide insight into how sister chromosomes are arranged during development of the *par* phenotype. By 4 hours after Topo IV inactivation, cells appear elongated with large unsegregated nucleoids, usually with one or two closely spaced *gln* foci at midcell (Figure 4D). This phenotype is maintained for longer 42°C incubations (data not shown), and cells eventually arrest growth with multiple half-segregated chromosomes (see two-color FISH labeling, Figure S5).

### Genetic interactions between Topo IV and SeqA

**Figure 5:**
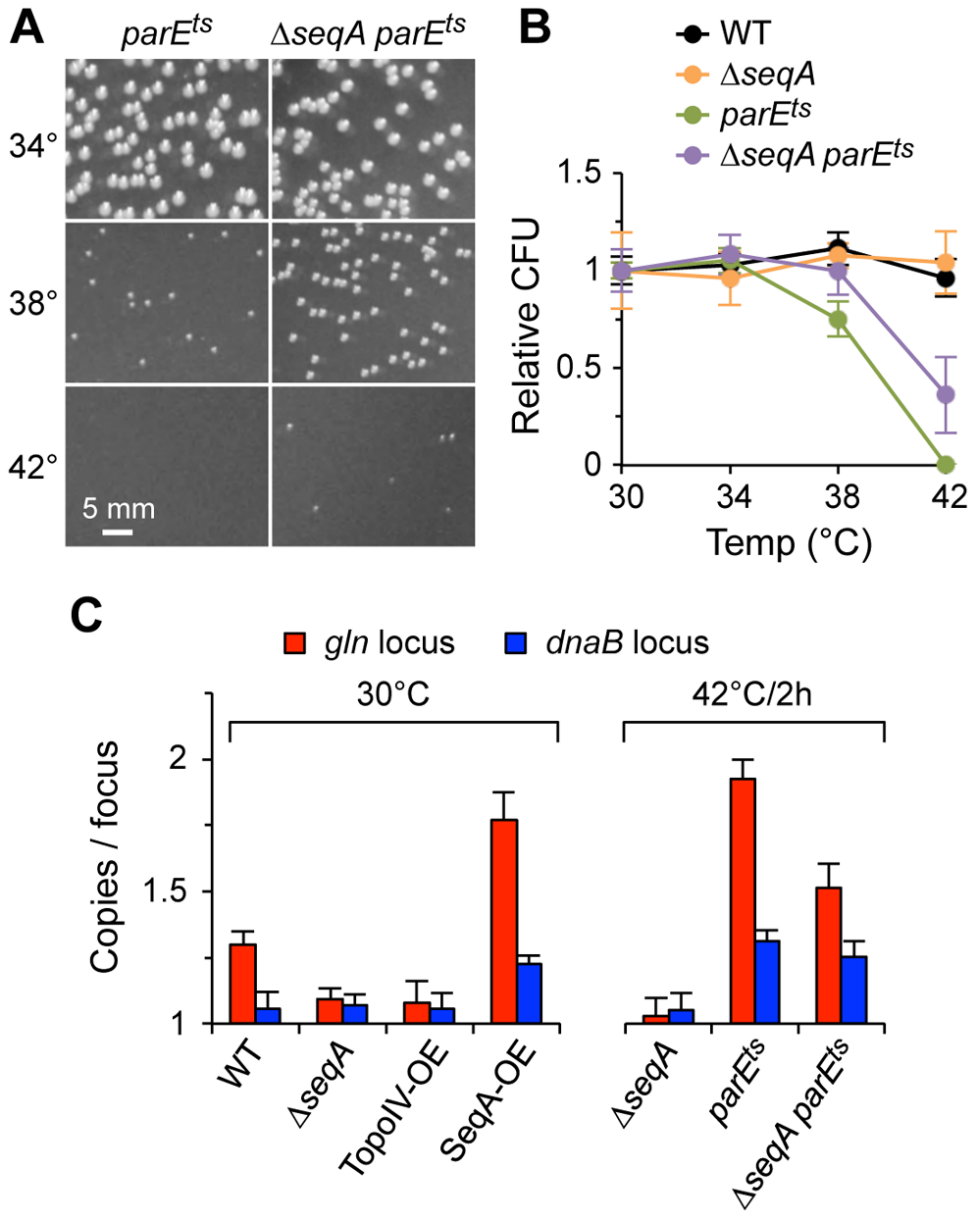
Genetic interactions between Topo IV and SeqA. (A) *ΔseqA* partially suppresses the temperature sensitivity of *parE10*. *parE10* single and Δ*seqA parE10* double mutant cells were grown to exponential phase at 30°C, equal numbers of cells were plated and incubated at the indicated temperature for 24 h and colonies were photographed. (B) Quantification of colony formation data. Wild-type, Δ*seqA* and *parE10* single and double mutants were plated and grown as described above, and the number of colonies relative to WT grown at 30°C were plotted (2 independent experiments ±1 SD). (C) Cohesion relationship between SeqA and Topo IV. Cohesion values were determined at *gln* and *dnaB* in cells carrying loss-of-function alleles or overexpression constructs of *seqA* and *parE*. Overexpression (OE) was achieved by transformation with low-level expression plasmids (Materials and Methods). Vector plasmids showed wild-type cohesion levels at *gln* and *dnaB* (1.31±0.07 and 1.05±0.04 copies/focus, respectively).

To determine the epistatic relationship between Topo IV and SeqA, we tested a *parE10* Δ*seqA* double mutant for temperature sensitivity and cohesion. Single mutant *parE10* cells exhibited partial growth at 38°C, with ∼25% reduction in colony forming units (CFU) compared to 30°C, and no growth at 42°C (Figure 5A; 5B, green), while Δ*seqA* single mutants (orange) grew well at all temperatures. Double mutant *parE10* Δ*seqA* cells (purple) showed intermediate growth at both 38°C and 42°C, indicating partial suppression of the *parE10* Ts phenotype. Double mutant *parE10 ΔseqA* cells showed ∼40% decreased cohesion compared to *parE10* alone, but suppression by Δ*seqA* was specific to *gln* (Figure 5C right, red bars); elevated *dnaB* cohesion in *parE10* at 42°C was not significantly reduced by addition of Δ*seqA* (blue bars). The relationship between SeqA and Topo IV was further examined by mildly overexpressing each protein from a low copy inducible expression vector and testing *gln* and *dnaB* cohesion (Figure 5C left). Cells induced for Topo IV expression (Topo IV-OE) for one hour prior to observation had significantly reduced *gln* cohesion, to a level similar to that seen in Δ*seqA* (red bars). Conversely, cells overexpressing SeqA protein (SeqA-OE) had the opposite phenotype, with >2-fold increase in *gln* cohesion and *dnaB* cohesion, similar to *parE10* cells at 42°C. This phenotypic similarity also extended to cell morphology; Δ*seqA* and Topo IV-OE cells had poorly separated nucleoids and closely spaced *gln* foci, while SeqA-OE and *parE10* cells were very elongated often with one mid-cell *gln* focus (example, Figure S6). Topo IV expression was normal in *ΔseqA* cells (Figure S7) as shown previously [33].

Because the cohesion phenotype of a *parE10 ΔseqA* double mutant most closely resembled that of a *parE10* single mutant, the simplest interpretation of the above results is that *parE* is epistatic to *seqA* (SeqA acts upstream of Topo IV in a single pathway). Although this conclusion assumes complete penetrance of the *parE10* mutation (no partial or compensating activity at 42°C), it is supported by the fact that Topo IV overexpression was able to reduce cohesion levels well below wild-type, even in the presence of SeqA protein, indicating that all cohesion probably occurs via a precatenane mechanism (Discussion). Cohesion along snap regions is apparently more complicated, where it is clear that SeqA has some Topo IV-independent function (Δ*seqA* reduced *gln* cohesion ∼40% in a *parE10* background). Such function could be direct bridging of sister chromosomes or negative regulation of compensating topoisomerases (Gyrase or Topo III).

### Cohesion is required for efficient chromosome segregation

**Figure 6:**
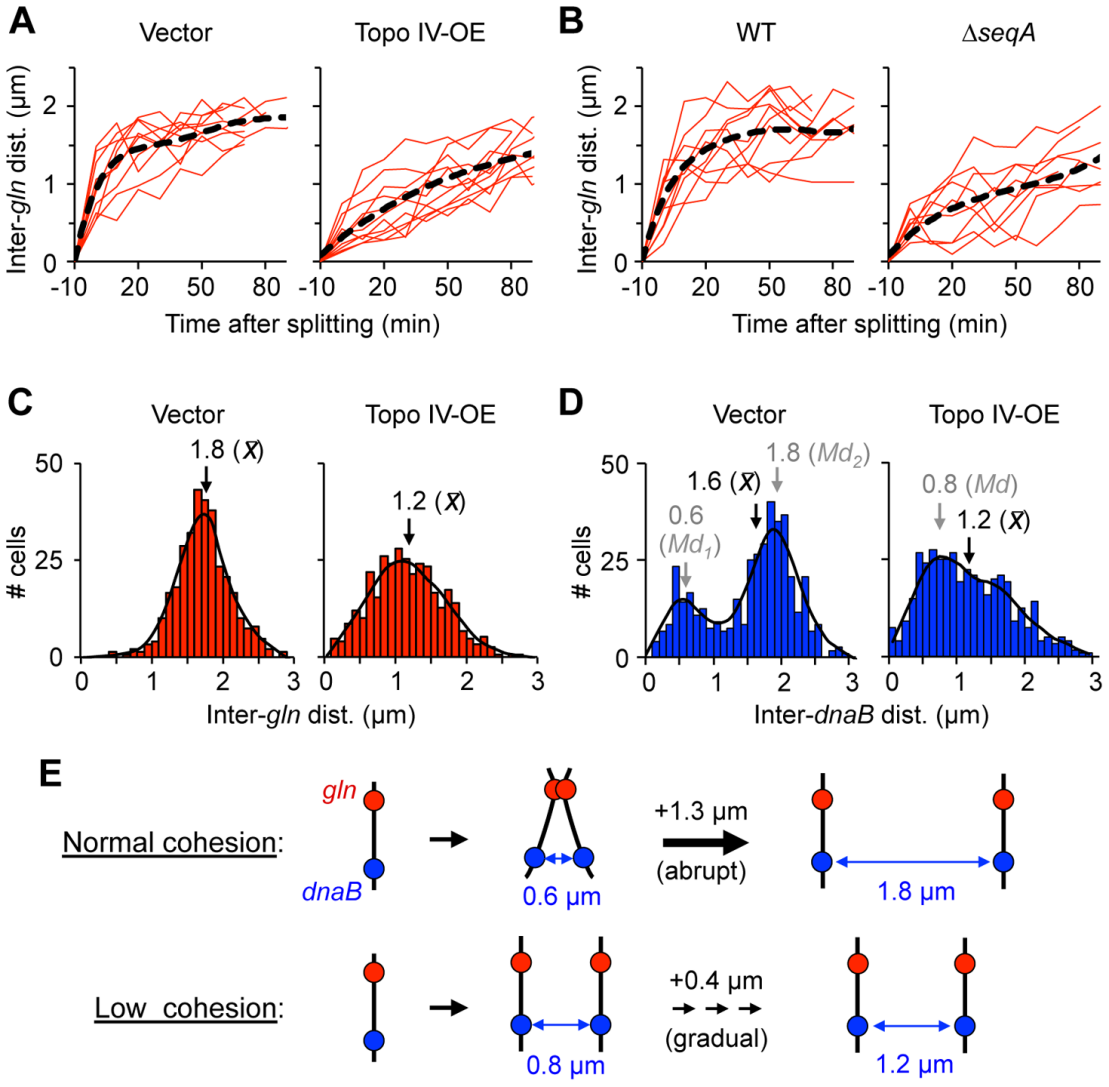
Cells with reduced cohesion have impaired chromosome segregation. (A) Separation velocity is reduced in cells overexpressing Topo IV. Cells bearing a *tetO* array at *gln* and expressing TetR-mCherry were transformed with pDB332, a weak Topo IV expression plasmid (Topo IV-OE), or the empty vector pBAD322-kan (Vector), grown to log phase in AB succinate +CAA, induced for two hours, placed onto agarose pads and imaged by time-lapse microscopy. Inter-focus distance was measured every 10 minutes through one cell cycle. Time zero represents the first time point at which two foci appeared. Regression line (black) is shown for 10 independent cells (red). (B) Separation velocity is reduced in Δ*seqA* cells. Cells were grown and analyzed as above. (C–D) Topo IV overexpressing cells exhibit abnormal *gln* (C) and *dnaB* (D) focus positions. Inter-focus distances were determined in exponentially growing cells by FROS. Regression line (black), and mean (x) and mode (Md) inter-focus distances are shown for each histogram (n = 500). (E) Graphical summary of *gln* and *dnaB* segregation data in cells with normal or reduced cohesion (see Text for details).

Coordinated separation of *gln* and four other late-splitting snap loci on the right chromosome arm is accompanied by a 35% increase in nucleoid volume and deformation of the nucleoid into a bi-lobed mass with one copy of each replicated sequence positioned within each lobe [6]. This suggests that cohesion loss along these tightly cohered regions initiate and/or drive a key sister individualization step in *E. coli* chromosome segregation. We tested this hypothesis by measuring the rate of separation of segregating sister loci in cells with reduced cohesion after genetic manipulation of SeqA or Topo IV. Time-lapse analysis of *gln* segregation was performed by growing and imaging cells directly on agarose-coated slides. Cells bearing a *tetO* array at *gln* and a photostable TetR-mCherry fusion that allowed multiple (10–15) exposures were imaged every 10 minutes through one complete doubling time (2 h). Under these conditions, the majority of cells underwent a single round of replication per cell cycle and contained either one or two *gln* foci (data not shown). Control cells exhibited abrupt *gln* separation with an average inter-*gln* distance of ∼1 µm immediately after appearance of two *gln* foci (Figure 6A, left panel, t = 0). Inter-*gln* distance continued to increase to ∼1.5 µm by 20 minutes after splitting, then gradually increased to a maximum of ∼2 µm before cell division. This corresponds to an initial separation speed of ∼0.15 µm/min, slowing to the rate of cell elongation (∼0.02 µm/min) by 20 minutes after focus duplication (Figure S8A). When images were acquired every 3 minutes, split *gln* foci still initially appeared ∼1 µm apart, indicating that the actual speed of focus separation likely exceeded 0.4 µm/min (Figure S8B; Movie S1). This estimation is in line [9] or slightly higher [34] than previous measurements.

In cells overexpressing Topo IV for one hour before imaging, *gln* separation was much slower, with split *gln* foci initially appearing ∼0.4 µm apart (Figure 6A, right panel) and separation speeds ∼1/3 of that seen in non-overproducing cells (Figure S8). Supporting the time-lapse data, inter-*gln* distance after Topo VI overexpression in exponentially growing batch culture cells (n = 500) was significantly reduced, with a wider distribution compared to vector control cells (Figure 6C). Similarly to Topo IV overexpression, Δ*seqA* cells showed protracted *gln* segregation with ∼70% decrease in initial *gln* separation velocity compared to WT (Figure 6B, right panel; Movie S2). This finding implies that cohesion specifically along late-splitting snap regions is required for efficient chromosome segregation.

An equally pronounced effect of Topo IV overexpression was seen on the distribution of inter-*dnaB* foci (Figure 6D). The non-snap *dnaB* locus normally exhibits a bimodal distribution of inter-sister distances corresponding to times before and after snap separation [6]. This pattern, which was seen in vector control cells (Figure 6D, left panel), implies that *dnaB* segregation occurs in two discrete steps: an initial separation to 0.6 µm apart, followed by a second larger separation event (to 1.9 µm) later in the cell cycle (Illustrated in Figure 6E). Other non-snap loci behave similarly, and we have proposed that early separation of these loci is restrained by long-lived connections along snap regions [6]. After cohesion reduction by overexpression of Topo IV, this bi-modal positioning was lost, and sister *dnaB* loci had a ∼25% lower average inter-focus distance than vector control cells (Figure 6D, right panel). We conclude that reduced cohesion causes inefficient segregation of both snap and non-snap loci.

## Discussion

An examination of cohesion in 8 chromosome structure and segregation mutants identified Topo IV and SeqA as strong and opposite mediators of sister chromosome cohesion in *E. coli*. Mutants deleted for the nucleoid associated proteins HU, IHF or Fis, all of which can condense DNA by bridging adjacent chromosomal segments [18], exhibited no detectable loss or gain of cohesion. Similarly, mutants of the SMC-like proteins RecN and MukB, structurally related to the cohesin proteins responsible for linking homologous chromosomes in eukaryotes, also had no effect on cohesion in our study. Depletion of Topo IV via a temperature-sensitive *parE10* mutation resulted in a rapid increase in sister cohesion at all loci, development of a large (multi-chromosome) unsegregated nucleoid, and eventual cell cycle arrest. Conversely, deleting *seqA* caused up to a 60% decrease in sister cohesion, with the largest decreases seen at loci that normally bind high levels of SeqA. Reductions in cohesion had adverse effects on chromosome segregation, including sluggish separation speeds and incomplete nucleoid division.

### Precatenanes occur universally along the *E. coli* chromosome

We and others have previously showed that most chromosomal loci experience a 7–10 minute delay between passage of the replication fork and separation beyond a resolvable (∼230 nm) distance [3], [6]. During this period of colocalization, homologous sequences physically interact [11], suggesting that similar to eukaryotic chromosomes, sisters are tightly juxtaposed during cohesion. At a fork speed of 700 nt/sec [6], this means that a 300–400 kb sliding window of tight sister cohesion occur behind each replication fork. Superimposed on this progressive cohesion program, *oriC* and two broad >100 kb segments on the right chromosome arm remain cohered for 20–30 minutes [3], [5], [6]. Late-splitting right arm loci, or *snaps*, are further distinct from *oriC* and the rest of the chromosome in that they separate in unison and concomitantly with appearance of bi-lobed nucleoids [2], [6].

**Figure 7:**
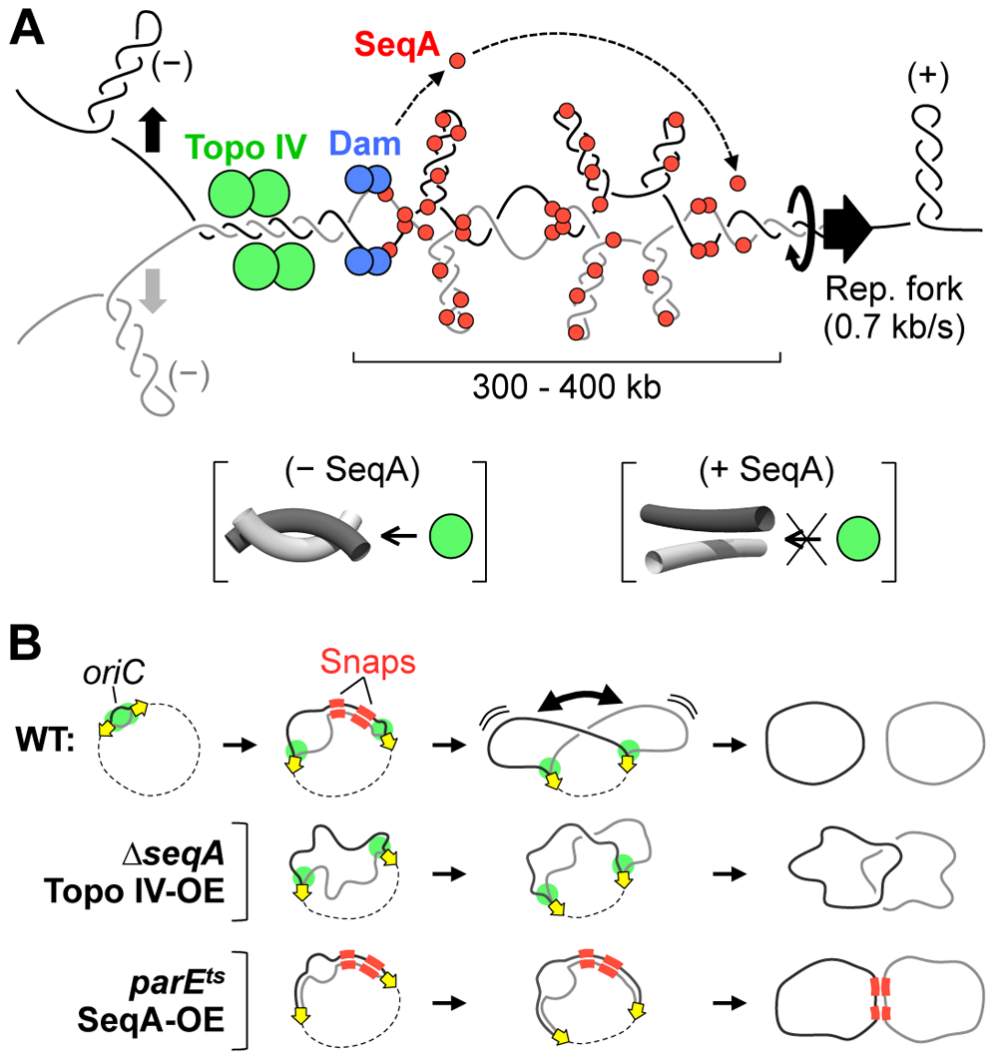
Models for cohesion and cohesion-mediated chromosome segregation. (A) SeqA-dependent precatenane removal. Positive supercoils migrate behind the replisome, entwining newly replicated sister regions. Resolution of precatenanes by Topo IV (green) is delayed by SeqA (red), which binds to hemimethylated DNA tracts behind the fork. Five to ten minutes after fork passage, DNA is remethylated by Dam (blue), releasing SeqA, and allowing Topo IV to resolve inter-sister links. SeqA may inhibit Topo IV by restraining supercoils, which transforms Topo IV-reactive hooked juxtaposition crossings (lower left brackets) to Topo IV-unreactive unhooked crossings (lower right brackets). (B) Simultaneous release of cohesion along right-arm snap regions (red) promotes abrupt sister separation and results in individualized daughter nucleoids (middle). Premature cohesion loss results in poor sister individualization (top). Deficient removal of cohesion results in late/unfinished sister separation (bottom).

Prior work by the Sherratt and Espeli labs indicated that segregation of *oriC* and *ter* sequences is modulated by Topo IV [4], [11]. Theoretically, duplex tension generated by the replicative helicases can migrate back behind the fork twisting nascent sister chromatids around each other as originally proposed by Cozzarelli and colleagues [35] (Figure 7A). Resolution of inter-sister twists, or precatenanes, requires a highly specific double strand cleavage, strand passage and ligation reaction that is mediated by the essential and highly conserved Topo IV protein [12]. Our current results extend the role of Topo IV to mediating cohesion of arm loci, including the late-splitting snap regions. Depleting Topo IV by shifting a *parE10* mutant to non-permissive temperature caused an immediate block of sister separation at all loci tested, resulting in the classic par phenotype of large undivided nucleoids in elongated cells (Figure 4). Conversely, overexpression of Topo IV resulted in dramatically reduced cohesion at all loci (Figure 5). From these data, it can be argued that precatenanes are the fundamental basis of all cohesive linkages in *E. coli*. Importantly however, precatenanes do not readily explain the phenomenon of late-splitting snaps. Although snaps are cohered 2–3 times longer than non-snap loci, both loci responded identically to loss of Topo IV (Figure 4C), indicating that high cohesion at snap loci is likely caused by another mechanism than Topo IV (below).

### SeqA delays separation of newly replicated DNA

Plasmid studies and *in vivo* estimates of decatenation kinetics indicate that the abundant Topo IV protein likely has a cellular precatenane unlinking capacity equal to or even faster than the rate that they are formed [16], [28], suggesting that additional factor(s) exist in *E. coli* to impede sister separation. We propose that SeqA protein fulfills this role by binding to the same newly replicated DNA stretches acted on by Topo IV. Null mutants of *seqA* exhibited significantly reduced cohesion, and this effect was strongest at sites containing a high local concentration of GATC sites. As predicted by this model, late-splitting snap loci and *oriC* have higher than average local GATC frequency and bind ≥10-fold more SeqA than non-snap loci by ChIP-qPCR. When SeqA is overexpressed, cohesion time increases >2-fold at both snap and non-snap loci, with a cohesion and nucleoid phenotype indistinguishable from *parE10* at 42°C (Figures 5,S6). We conclude that SeqA is the primary timekeeper for sister cohesion, and is solely responsible for extended cohesion observed along snap regions.

SeqA is uniquely suited to mediate sister cohesion due to its high specificity for hemimethylated DNA. Newly replicated strands are unmethylated for 5–10 minutes after passage of the replication fork before methylation by Dam [21], and SeqA-GFP fusions form large, relatively immobile foci adjacent to the replisomes [19], [22], [36]. In the absence of Dam, SeqA does not form these complexes [22], and in our study *dam*- cells exhibited a low cohesion phenotype identical to *seqA^−^* (Figure 1). Based on an average spacing of one favorable SeqA binding site per kb [19], an estimated 100–200 SeqA dimers are continually bound behind each fork (Figure 7A). Our data indicate that *oriC* and snaps bind several fold more SeqA than the more typical non-snap DNA, which may induce a higher order SeqA complex with increased stability [23], [37]. Given that overexpression of SeqA delays remethylation of origin DNA [38], and SeqA dimers can oligomerize into Dam-resistant RecA-like filaments along GATC-dense DNA fragments *in vitro* [23], [37], it is possible that SeqA binds as individual dimers along most of the chromosome and as a continuous or semi-continuous filament along snap regions.

### A two pathway model

Two models can explain how SeqA modulates sister cohesion. SeqA could stimulate cohesion directly by forming protein-protein linkages across sister chromosomes, or it could promote cohesion indirectly by inhibiting the activity of Topo IV along precatenated junctions. The best evidence for a direct mechanism is that double mutant Δ*seqA parE10* cells exhibited an intermediate cohesion phenotype to each of the single mutants. Although the *parE10* mutation was partially epistatic to Δ*seqA* (double mutants more closely resembled *parE10*), it is clear that at least part of SeqA's ability to promote cohesion was independent of Topo IV, suggesting that these proteins reside in different pathways. In support, purified SeqA has been shown to physically tether hemimethylated *oriC*-containing molecules in an *in vitro* replication system [36]. Additionally, the binding characteristics of SeqA dimers suggest that oligomerization may be facilitated by individual subunits binding across opposing homologous GATC sites as they exit the fork [37]. It is unclear how SeqA nucleoprotein complexes are eventually disassembled, but SeqA molecules have an on/off rate that exceeds the hemimethylation period [39], suggesting that another factor controls the lifespan of SeqA complexes, possibly Dam.

### A one pathway model

If increased cohesion at snap loci is indeed due to inter-sister bridging by SeqA, then overexpression of Topo IV would be expected to have little effect on snap cohesion in a *seqA* ^+^ strain. This was clearly not the case in our study: Topo IV overexpression resulted in a 3-fold reduction in cohesion at the *gln* snap locus, with cohesion and nucleoid phenotypes identical to Δ*seqA*. Similarly, SeqA overexpression phenocopied *parE10* at 42°C. The simplest interpretation of these results is that SeqA and Topo IV reside in the same pathway, with SeqA inhibiting Topo IV decatenation. Observed partial synergism between Δ*seqA* and *parE10* (two pathway, above) could result from SeqA inhibiting DNA gyrase or Topo III, which are known to partially compensate for Topo IV function at the replication fork [15].

How could SeqA inhibit precatenane removal? If present in sufficient quantities, SeqA could conceivably physically block access of Topo IV to catenated structure. However this may be unlikely given that Topo IV binding is not sequence-specific, and typical DNA exhibiting ∼10 minutes of cohesion contain only sparse (∼one per kb) SeqA binding sites [19]. Instead, we favor a topological-based mechanism in which SeqA binding temporarily sequesters positive supercoils behind the fork, preventing Topo IV from recognizing catenated DNA crossings. Normally in positively supercoiled DNA, duplex crossings (inter or intra-molecular) adopt a tight geometry with signature “hooked juxtapositions” (Figure 7A) that are recognized and cleaved by Topo IV and gyrase [40]. This mechanism may explain how Type-II cleavage, strongly cytotoxic if unregulated and used as a chemotherapeutic, is limited to only positively supercoiled regions [16], [40]. SeqA binding, which is known to alter DNA twist or writhe by restraining supercoils [23], might relax inter-sister junctions, preventing Topo IV-mediated decatenation (Figure 7A). Further, SeqA has been shown to directly modulate Topo IV-mediated cleavage *in vitro*, inhibiting decatenation at high SeqA concentrations and favoring decatenation at lower concentrations [24]. We speculate that variable binding of SeqA along the *E. coli* chromosome results in a wide dynamic range of Topo IV regulation, and may explain the highly variable and “patchy” behavior of sister cohesion. An analogous mechanism may operate in eukaryotes, in which the ring-like cohesin complex retards decatenation of sister chromosomes by inhibiting topoisomerase II, the eukaryotic homolog of bacterial Topo IV [41].

### Chromosome snaps mediate efficient chromosome segregation

In wild-type cells, sister snap segregation is very rapid, with foci appearing 1.5 µm apart within 20 minutes after splitting, and separating with an initial velocity of ≥0.4 µm/min. Repressing cohesion via a *seqA* deletion or overexpression of Topo IV, resulted in 30% reduced final inter-sister distances and 70% slower initial separation velocities (Figures 6,S8). Although we hypothesize that the observed segregation defects in these strains were a direct consequence of reduced cohesion, it is possible that they were caused instead by effects on cell cycle timing or nucleoid compaction. For example, Δ*seqA* cells initiate prematurely [20], which could potentially advance segregation timing beyond its normal cell cycle window. They also exhibit over-condensed nucleoids, which might reflect some inability to separate newly replicated regions from the replisome [19]. However, replication timing defects in Δ*seqA* cells are suppressed under slow growth conditions [20], and replication initiation was only five minutes earlier than WT in our experiments (Figure 2A). Moreover, Topo IV overexpression, which has no known effect on the timing of DNA replication, resulted in slowed sister segregation that was indistinguishable from Δ*seqA*. In sum, we conclude that poor segregation in these strains was a direct result of reduced sister cohesion.

These findings provide direct supporting evidence for a previously proposed model in which snaps mediate a key mid-replication chromosome reorganization event (Figure 7B; [6]). This event involves the following coordinate chromosome transformations: 1) simultaneous release of inter-sister linkages along both snap regions, 2) conversion of the nucleoid from unilobed to bilobed morphology, 3) 35% increase in total nucleoid volume, 4) further dramatic separation of replicated non-snap loci, and 5) placement of one copy of each thus-far replicated sequence in each daughter nucleoid lobe. The net effect of these changes are conversion of the nucleoid from a highly condensed mixed state to a relaxed pre-divisional state with spatially individualized sister chromosomes (Figure 7B). Recent work from the Kleckner lab has shown that *E. coli* progresses through four chromosome expansion stages (T1–T4), with the above described T2 transition being the most prominent in terms of sister separation [7].

How could holding sisters together promote their separation? Cohesion at eukaryotic centromeres directs sister chromatid segregation by providing counter tension between opposing microtubule assemblies. Similarly, it is possible that snaps resist global separation of replicated *E. coli* chromosomes until they are acted on by an ‘external’ segregation mechanism such as MukB [42], FtsK or MreB. Or, in theory, pushing forces generated between highly confined snap segments during cohesion, and their simultaneous release, could drive sister separation without outside influence [7]. Such cycles of restraint and programmed release of DNA confinements are proposed to be a general basis for chromosome movements observed in eukaryotes [43]. In fact, release of cohesion along chromosome arms in prometaphase is required for the generation of compact side-by-side sister chromatids long before microtubule involvement ([43] and references therein). Given that identified snap regions comprise only a small fraction of the total genome, we speculate that snap splitting is a triggering mechanism for a global nucleoid reorganization event that relies on a combination of internally and externally derived forces. Despite dramatically slowed sister separation velocities in the absence of cohesion, most cells were eventually able to complete chromosome segregation, with moderate cell elongation and production of anucleate cells (e.g., Figure 3B). Thus, the significance of cohesion in the greater *E. coli* chromosome segregation program remains somewhat clouded. It is logical to assume that segregation defects in cohesion-less cells observed under the current slow growth conditions are compounded during multi-forked replication, which is in agreement with the rich media sensitivity of Δ*seqA* strains [20], [30].

### SeqA, the multi-faceted genome stability factor

SeqA plays a prominent role in nearly every phase of genome duplication and inheritance. First discovered in a screen for mutations that allowed replication of a hemimethylated *oriC* plasmid [44], SeqA binds and sequesters *oriC* immediately after replication starts for about one third of the replication period, during which *oriC* is refractory to further initiations [21], [44]. There is also evidence that SeqA stabilizes replication fork progression: *seqA* mutants grown in rich medium exhibit stalled replication forks after rifampicin runoff [20], and they are hypersensitive to the replication elongation inhibitors hydroxyurea (HU) or azidothymidine (AZT) [45]. SeqA's ability to organize replication forks (or at least the DNA created by forks) into so-called “hyperstructures” is well documented [19], [22], [36]. This activity has been hypothesized to improve fork progression by concentrating replication proteins to a central location [36], [46] and even to drive chromosome segregation by continually condensing daughter nucleoids on either side of the replisome [19]. Our current work shows that SeqA promotes sister cohesion, and that extended cohesion along snap regions is involved in a global chromosome reorganization event that is important for efficient chromosome segregation. Through its capacity to indefinitely cohere DNA, SeqA may also mediate cell cycle blockage during the stringent response, as indicated by a requirement of SeqA for nutritional deprivation-induced chromosome segregation blockage, independently of its function at *oriC* [45]. Logically, cohesion in *E. coli* may also drive homologous recombination dependent DNA repair by co-localizing sister molecules immediately after replication, presumably when double strand breaks are created. Supporting this model, *seqA* mutations result in mild SOS induction [33], [47], and are synthetically lethal with *recA* mutations in rich medium [47].

## Materials and Methods

### Bacterial strains and growth conditions

The genetic background for all strains is DB81, a derivative of CM735 (*metE46 trp-3 his-4 thi-1 GalK2 lacY1, lacZ4 mtl-1 ara-9 tsx-3 ton-1 rps-8*, or *rps-9 supE44* lambda) [48] containing the *Ptac*-*fliC^st^* synchronization allele [29]. Gentamycin-marked *tetO* array insertion strains were previously described [6]. Gene deletion or disruption alleles were obtained from the following sources: *ΔseqA* in-frame deletion [44]; *dam13*::*Tn9* [49]; *parE10* and *parE1215* [27]; *hupA*::*cat* and *hupB*::*kan* [50]; *mukBEF*::*kan* [51]; *fis767*::*kan* [52]; and *ihfA*::*cat* [53]. Marked alleles were introduced into DB81 by P1 transduction selecting for antibiotic resistance or in the case of *parE10* and *parC1215* reversion of methionine auxotrophy, *ΔseqA* was introduced by the gene replacement vector pBIP [44]. Cells were grown in AB minimal media supplemented with 0.2% alanine and 20 µg/ml each of tryptophan, histidine, methionine and thiamine or 0.2% succinate and 0.1% casamino acids, as indicated. These media resulted in doubling times for DB81 at 30°C of 126 minutes and 83 minutes, respectively. Cell synchronization was carried out as previously described [6].

### Fluorescence microscopy and analysis

All images were acquired with a Zeiss AxioImager Z1 microscope equipped with a Hamamatsu EM-CCD camera, and FROS and FISH data was analyzed using a custom Matlab image analysis program, FocusCounter (http://www.bcm.edu/genetics/bateslab). Raw foci/cell values were adjusted for focus detection inefficiency, determined empirically for each experiment based on the frequency of cells with zero foci (Figure S2). Detection inefficiencies ranged between 0.9% and 3.6% (avg. 1.4%, ±0.8%), resulting in final corrections of only +0.06 to +0.15 foci/cell. This method was validated by accurately calculating foci/cell in a control experiment with cohesion-less stationary phase cells (Figure S2).

FROS was performed as previously described [6]. Cells carrying the TetR-YFP expression plasmid pDB316 were grown to OD 0.2 with 50 ng/ml ampicillin, induced with 0.02% arabinose for 1 hour, then imaged directly without fixation. pDB316 is a derivative of pWX6 [54] that carries a deletion of the LacI-CFP gene and a spontaneous mutation that weakens expression. For time-lapse experiments, TetR-mCherry was expressed from pDB317, a derivative of the salicylate-inducible *nahG* promoter vector pKG110 that provides highly tunable expression at sub-micromolar concentrations of inducer. Cells were grown to OD 0.2 with 25 µg/ml chloramphenicol and 50 µg/ml anhydrotetracycline (to reduce TetR binding), induced with 0.5 µM sodium salicylate for one hour, placed onto agarose-coated slides (liquid media with 2% SeaKem ME low melting agarose) and imaged in a controlled temperature 37°C environment. Unless otherwise noted, ∼1000 cells are analyzed per sample for all experiments. An absence of replication pausing or blockage at the array site was confirmed by qPCR analysis for all FROS experiments (Figure S9AB). Such blocks can occur under high TetR expression and was observed with the original TetR expression plasmids pLAU53 or pWX6 ([54]; Figure S9). Additionally, when cohesion was analyzed in cells without a *tetO* array by FISH, copy number and foci per cell in both WT and *parE10* strains were very similar to values obtained by FROS (Figure S10).

For FISH, DB81 cells (*parE10* and *parE+* derivatives) without *tetO* array were grown to exponential phase in minimal succinate medium and fixed with 2.5% paraformaldehyde. Three kilobase *gln* and *dnaB* probes were amplified by PCR (Table S1) and labeled with PromoFluor-500 or -594, respectively by nick translation (Promokine, Germany). *In situ* hybridization was performed as previously described [6].

### Copy number and cell cycle determinations

The number of *gln* or *dnaB* loci per cell was determined by measuring the relative ratio of *gln* or *dnaB* loci to *oriC* loci by qPCR as previously described [6]. These ratios were then multiplied by the total number of *oriC* loci per cell determined by Rifampicin runoff analysis of duplicate cell samples (Figure S1). To exclude any error caused by possible rifampicin-resistant initiations in mutant cells (e.g., [53]), *gln* and *dnaB* copy numbers were verified by absolute quantification qPCR in which cell samples were spiked (1∶1) with a calibrator strain containing a unique sequence that was used to generate a standard curve of DNA copies per cell (values were ±0.08 of those shown in Figure 1). Real time qPCR was performed in 384-well plate in ABI Prism 7900HT Thermal Cycler using KAPA SYBR Fast qPCR reagent (Kapa Biosystems, USA) and analyzed with ABI-prism software (primers in Table S1).

Cohesion timing at a given locus in exponential cultures is measured as the ratio of locus copy number to foci per cell (Figure 1A), thus cell cycle determinations are not required. In synchronized cell experiments (Figure 2), cohesion duration is determined directly by measuring the timing of locus replication and segregation [6]. Locus replication time is equal to the point at which 50% of cells have duplicated locus copy number by qPCR (the replication cumulative curve), and locus segregation time is the point at which 50% of cells have duplicated the number of foci per cell (the segregation cumulative curve). Similarly, the timing of replication initiation and termination are equal to the point at which 50% of cells duplicate the *oriC* and *ter* loci, respectively. Resulting B, C and D periods (Figure 2B) are generated from the above replication timing and generation time.

### Overexpression studies

For Topo IV overexpression, the *parC* and *parE* open reading frames were amplified from the chromosome by PCR with *Eco*RI and *Hind*III restriction tails at 5′ and 3′ ends (Table S1) and cloned into pBAD322-kan [55], a low copy arabinose-inducible vector designed to express genes that are toxic at high levels. The resulting plasmid, pDB332, modestly overexpressed Topo IV after two hours induction with 0.02% arabinose (6-fold over WT; Figure S7), did not impede growth or cause cell filamentation after many generations of growth, and completely suppressed temperature sensitivity of both *parE10* and *parE1215* alleles (data not shown). This suggests that Topo IV overexpression did not block chromosome segregation or create DSBs, which could bias chromosome segregation analyses. For SeqA overexpression, the *seqA* ORF-containing *Bss*HII fragment (excluding the downstream *pgm* gene) was cloned into the expression vector pGC2 under Plac promoter control and containing the *lacI^Q^* fragment to reduce leaky expression, resulting in pDB338. Induction of pDB338 containing cells with 25 nM IPTG for 2 hours did not exhibit decreased DNA synthesis by flow cytometry (data not shown), as can occur under high SeqA expression [38].

### ChIP-qPCR

Three copies of the haemagglutinin (HA) epitope (TACCCATACGACGTCCCAGACTACGCT) were cloned onto the 3′ end of *seqA*, and integrated into the endogenous DB81 *seqA* locus via pBIP gene replacement. The resulting SeqA-HA_3_ protein exhibits a *seqA*+ phenotype as shown by normal growth rate and synchronous replication initiations (Figure S11). Chromatin immunoprecipitation was performed essentially as in [32]. Briefly, DB81 *seqA-HA_3_* cells were grown in AB alanine media to early log phase, formaldehyde cross-linked, lysed, and sonicated to fragment DNA. Triplicate samples of cross-linked SeqA-HA_3_-DNA were immunoprecipitated with monoclonal 12CA_5_ anti-HA antibody (Roche). Samples were washed, cross-links were reversed, and DNA was purified. Total DNA was also prepared from identical control “input” samples not subjected to immunoprecipitation. The relative abundance of five different sequences of bound DNA was determined by qPCR using specific primer pairs previously described [6]. For each input and IP DNA sample, qPCR was performed in triplicate and amplification Ct values were averaged. Fold-enrichment of bound DNA at each site was determined by the ΔΔCt method, where ΔΔCt equals the difference in amplification of IP DNA and input DNA for each site relative to *dnaB* (the locus showing lowest abundance in IP samples). Thus, ΔΔCt_sitex_ = (Ct_IP_−Ct_input_)_site*x*_−(Ct_IP_−Ct_input_)*_dnaB_*, and fold-enrichment = 2^−ΔΔCt^.

## Supporting Information

Figure S1 Rifampicin runoff histograms in mutant strains. Rifampicin and cephalexin were added to exponentially growing cells, which were allowed to complete ongoing rounds of replication (runoff). Cells were fixed in 70% ethanol and stained with 50 µg/ml DAPI, then analyzed on a BD Biosciences LSR Fortessa cytometer. Peaks corresponding the number of origins per cell at the time of drug addition were quantified and average origin per cell values were multiplied by the relative frequency of *gln* per *oriC* (0.98) from qPCR to give *gln* copy number (Figure 1A).
(TIF)

Figure S2 Correction of foci counts for detection inefficiency. Detection of fluorescent foci in our current FROS system is 96.4–99.1% efficient, thus foci per cell measurements are ∼2% lower than the true values of segregated loci. For each experiment, the detection efficiency is calculated from the percentage of cells with zero foci. Most zero-focus cells arise either from an absence of TetR-YFP expression (eliminated from the analysis) or from cells which expressed TetR-YFP but suffered from poor detection. (A) Cells that fail to express TetR-YFP are detected by low cell background fluorescence and eliminated from further analysis. Average cellular fluorescence was determined in non-focus regions in cells bearing a *gln tetO* array and induced for TetR-YFP expression. Pixel intensities are shown for three representative cells with zero (blue), one (green) or two (red) foci, as well as slide background (black). (B) Histogram of average fluorescence intensity for 100 measurements of each type to illustrate selection of TetR-YFP negative cells (normally 2–8% of cells in a field). (C) FROS accurately measures the number of *gln*'s in cohesion-less cells. Stationary phase cells (which presumably have fully replicated and segregated chromosomes) were assayed by FROS, and foci per cell values were corrected for detection inefficiency (IE) based on the percentage of YFP-positive cells with zero foci ([6]; Materials and Methods). Corrected foci/cell value matches the numbers of chromosomes per cell determined by flow cytometry.
(TIF)

Figure S3 High-resolution analysis of SeqA Chip-chip data. SeqA binding near four regions of interest is shown by plotting a 5-kb moving average of SeqA Chip-chip (log2 ratio of IP to input fluorescence) from Waldminhaus et. al. [31]. Plots indicate binding along 5 kb of chromosomal DNA centered on each *tetO* insertion site (Figure 3C,D). Note that *oriC* data is shown on a different scale.
(TIF)

Figure S4 Continued replication in the absence of Topo IV. (A) Wild-type and *parE10* cells from Figure 3A were analyzed by rifampicin runoff for 4 hours after temperature upshift. (B) DNA synthesis was measured in wild-type, *parE10* and *parE10* cells containing 0.5 mg/ml novobiocin, which targets Topo IV and DNA gyrase [28], by steady state radioactive thymidine incorporation.
(TIF)

Figure S5 Two-color FISH analysis of *gln* and *dnaB* in *parE10* cells. *parE10* cells were grown to exponential phase in minimal succinate media at 30°C, shifted to 42°C for 2 hours, then analyzed by two-color FISH at *gln* and *dnaB* loci (Materials and Methods). The majority of cells contained fewer *gln* foci than *dnaB* foci, indicating greater cohesion at *gln* in the absence of Topo IV (±1 SD of 3 independent experiments, 300 cells each).
(TIF)

Figure S6 Nucleoid and *gln*-YFP micrographs in cells deficient in or overexpressing SeqA and Topo IV. Representative cell images from Figure 5C are shown.
(TIF)

Figure S7 Topo IV expression analysis. Western blot analysis of Topo IV levels in WT, Δ*seqA*, and cells overexpressing Topo IV. Equal amounts of total protein (2 µg/ml) were loaded per lane. Blot was probed with monoclonal mouse anti-ParE (a kind gift of Lynn Zechiedrich) at 1∶10,000, detected with anti-mouse horseradish peroxidase, and imaged and quantified on a Storm Phosphorimager. Normalized intensities of ParE band relative to WT are shown. Topo IV overexpression was carried out using pDB332 as described in Figure 6.
(TIF)

Figure S8 Segregation velocities in cells overexpressing Topo IV. Focus separation velocity was assessed at *gln* by FROS timelapse in Topo IV overexpressing (Topo IV-OE) or control (Vector) cells as described in Figure 6. (A) Change in rate of segregation of sister loci (net positive separation between consecutive time points) was plotted for the 10-minute interval movies shown in Figure 6A. (B) Three-minute interval movies were acquired through the first 15 minutes after focus splitting and sister separation was plotted as above.
(TIF)

Figure S9 Current FROS system does not cause replication roadblocks. (A) Replication fork pausing at repressor-bound array sites was determined by qPCR analysis of segments immediately upstream and downstream of the array insertion. (B) Upstream∶downstream ratios ≈1.0 in all FROS strains used in the current study indicate an absence of replication pausing at the array site. For comparison, cells expressing TetR-YFP from pWX6 [54] in the absence of anhydrotetracycline (left-most column) have a >3-fold increase in DNA upstream of the array, indicating significant fork blockage.
(TIF)

Figure S10 Confirmation of FROS cohesion timing results with FISH. Wild-type and *parE10* cells without a *tetO* array were grown and analyzed as described in Figure 4, except that foci (segregation) were assayed by FISH (Materials and Methods). Results indicate that high cohesion in the absence of Topo IV is not an artifact TetR-YFP bound *tetO* arrays at the locus of interest. (A–B) Copy number per *gln* FISH focus (*A*) and per *dnaB* FISH focus (*B*) in *parE10* (dark shaded symbols) and *par^+^* control (light shaded symbols) cells after shift to restrictive temperature. Values are means of 3 experiments ±1 SD. (C) Relative cohesion at *gln* and *dnaB* in *parE10* cells.
(TIF)

Figure S11 SeqA-HA_3_ protein provides normal replication initiation. WT (DB81) and *seqA-HA_3_* strains were grown in LB at 37°C and assayed for origins per cell by Rifampicin runoff as in Figure S1. Synchronous replication initiation as shown by 2^n^ origins, indicates that HA-tagged SeqA is fully functional.
(TIF)

Movie S1 FROS timelapse of *gln*-YFP, vector control. Images were acquired every 3 minutes on agarose pads as described in Figure S7. Note focus brightness increases, presumably due to sister cohesion, then decreases at time of splitting. Bright foci occasionally separate into two foci then remerge (e.g., top cell).
(AVI)

Movie S2 FROS timelapse of *gln*-YFP, Topo IV overexpression. Images were acquired as above. Note slow separation of *gln* foci.
(AVI)

Table S1 Lists sequences for all cloning, qPCR and FISH probe primers.
(PDF)
