# Supplementary figures and images for: Regulation of Sister Chromosome Cohesion by the Replication Fork Tracking Protein SeqA
Source: PLoS Genet. 2013 Aug 22;9(8):e1003673. doi: 10.1371/journal.pgen.1003673 (PMC3749930; doi:10.1371/journal.pgen.1003673)

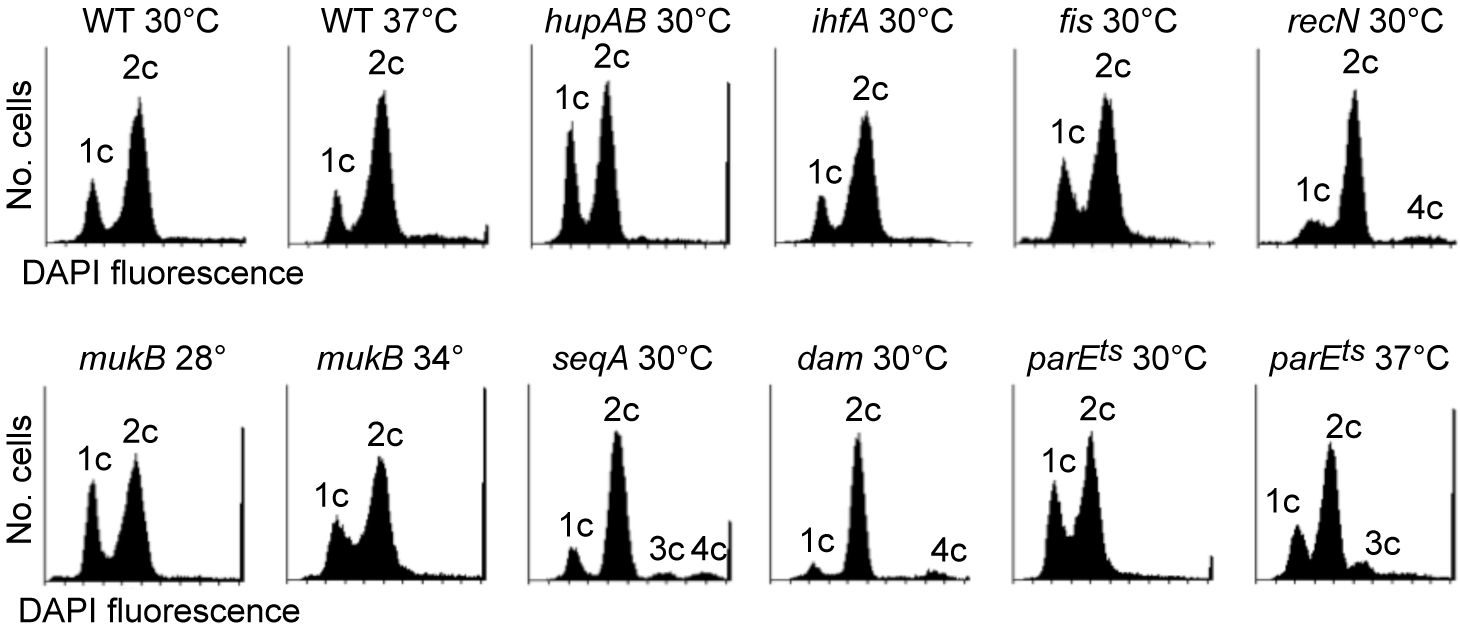

Supplement: Figure S1 — Rifampicin runoff histograms in mutant strains. Rifampicin and cephalexin were added to exponentially growing cells, which were allowed to complete ongoing rounds of replication (runoff). Cells were fixed in 70% ethanol and stained with 50 µg/ml DAPI, then analyzed on a BD Biosciences LSR Fortessa cytometer. Peaks corresponding the number of origins per cell at the time of drug addition were quantified and average origin per cell values were multiplied by the relative frequency of gln per oriC (0.98) from qPCR to give gln copy number (Figure 1A). (TIF) [file pgen.1003673.s001.tif]

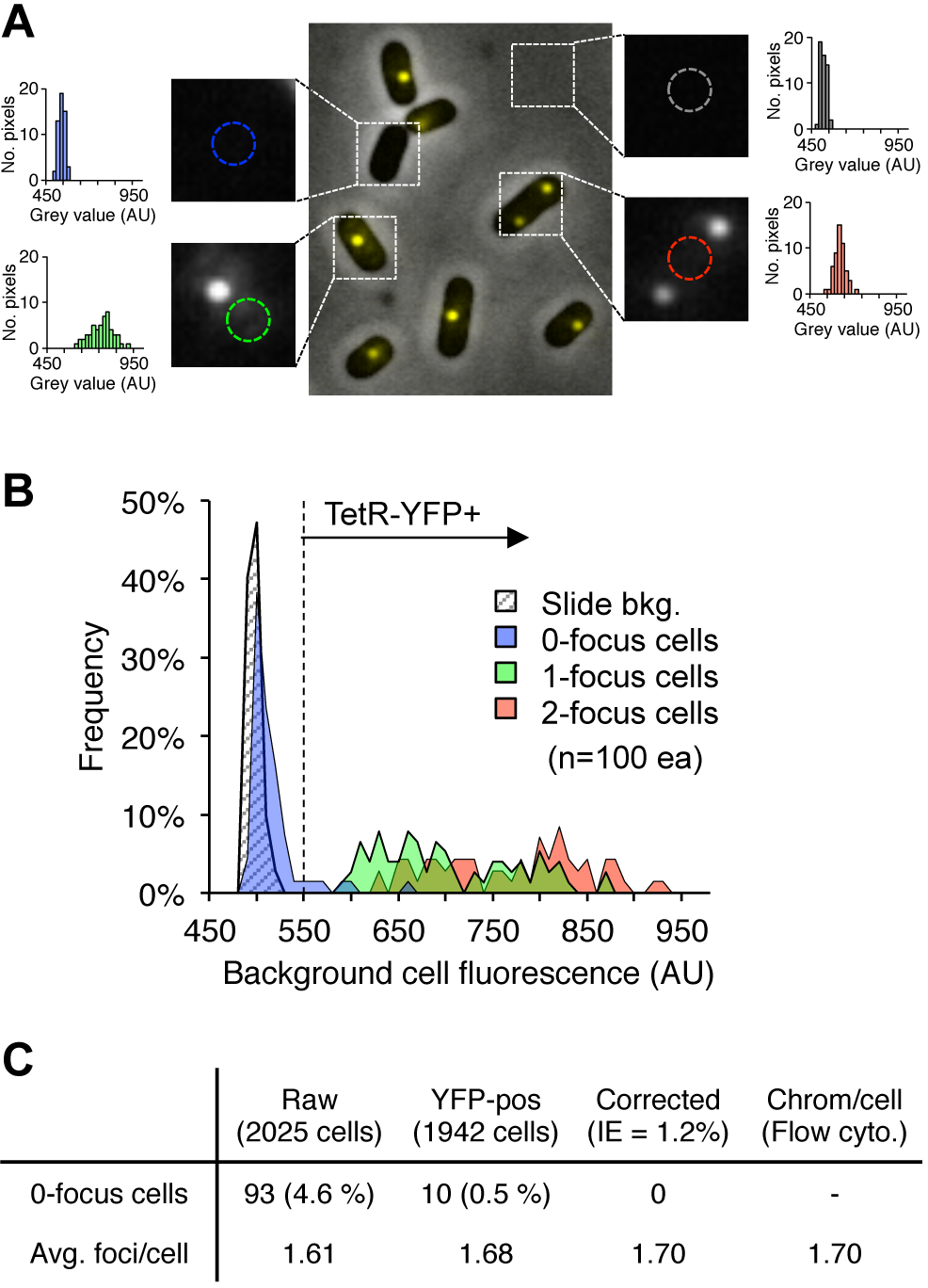

Supplement: Figure S2 — Correction of foci counts for detection inefficiency. Detection of fluorescent foci in our current FROS system is 96.4–99.1% efficient, thus foci per cell measurements are ∼2% lower than the true values of segregated loci. For each experiment, the detection efficiency is calculated from the percentage of cells with zero foci. Most zero-focus cells arise either from an absence of TetR-YFP expression (eliminated from the analysis) or from cells which expressed TetR-YFP but suffered from poor detection. (A) Cells that fail to express TetR-YFP are detected by low cell background fluorescence and eliminated from further analysis. Average cellular fluorescence was determined in non-focus regions in cells bearing a gln tetO array and induced for TetR-YFP expression. Pixel intensities are shown for three representative cells with zero (blue), one (green) or two (red) foci, as well as slide background (black). (B) Histogram of average fluorescence intensity for 100 measurements of each type to illustrate selection of TetR-YFP negative cells (normally 2–8% of cells in a field). (C) FROS accurately measures the number of gln's in cohesion-less cells. Stationary phase cells (which presumably have fully replicated and segregated chromosomes) were assayed by FROS, and foci per cell values were corrected for detection inefficiency (IE) based on the percentage of YFP-positive cells with zero foci ([6]; Materials and Methods). Corrected foci/cell value matches the numbers of chromosomes per cell determined by flow cytometry. (TIF) [file pgen.1003673.s002.tif]

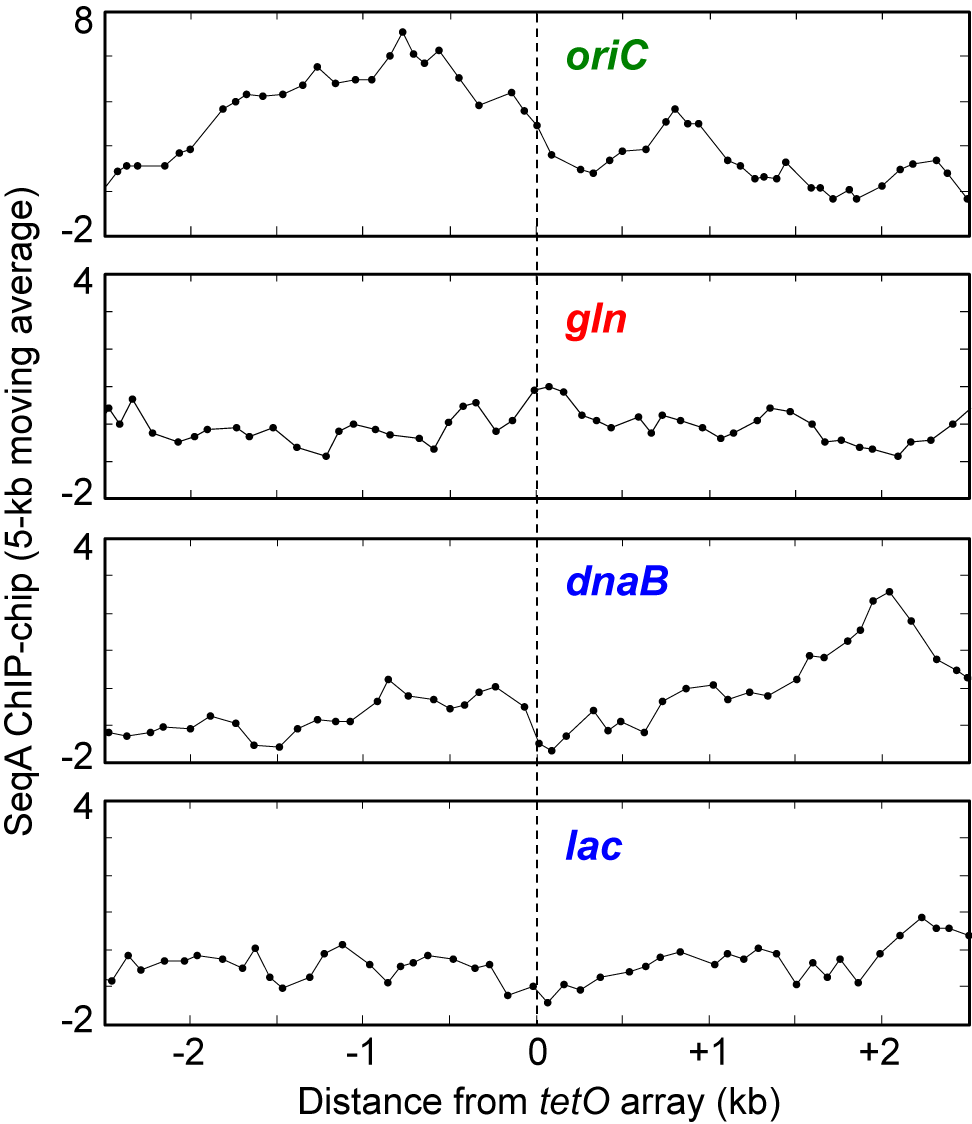

Supplement: Figure S3 — High-resolution analysis of SeqA Chip-chip data. SeqA binding near four regions of interest is shown by plotting a 5-kb moving average of SeqA Chip-chip (log2 ratio of IP to input fluorescence) from Waldminhaus et. al. [31]. Plots indicate binding along 5 kb of chromosomal DNA centered on each tetO insertion site (Figure 3C,D). Note that oriC data is shown on a different scale. (TIF) [file pgen.1003673.s003.tif]

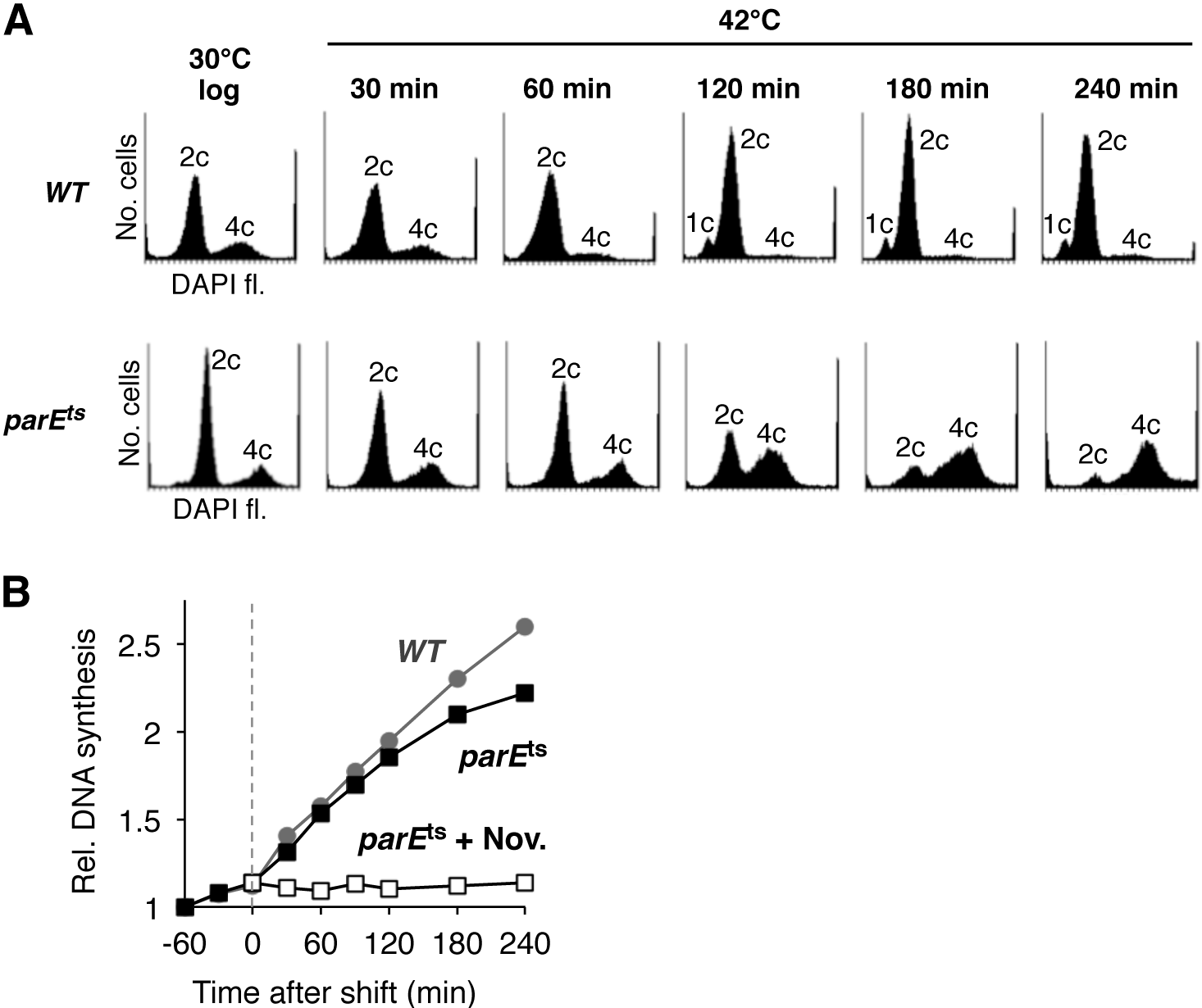

Supplement: Figure S4 — Continued replication in the absence of Topo IV. (A) Wild-type and parE10 cells from Figure 3A were analyzed by rifampicin runoff for 4 hours after temperature upshift. (B) DNA synthesis was measured in wild-type, parE10 and parE10 cells containing 0.5 mg/ml novobiocin, which targets Topo IV and DNA gyrase [28], by steady state radioactive thymidine incorporation. (TIF) [file pgen.1003673.s004.tif]

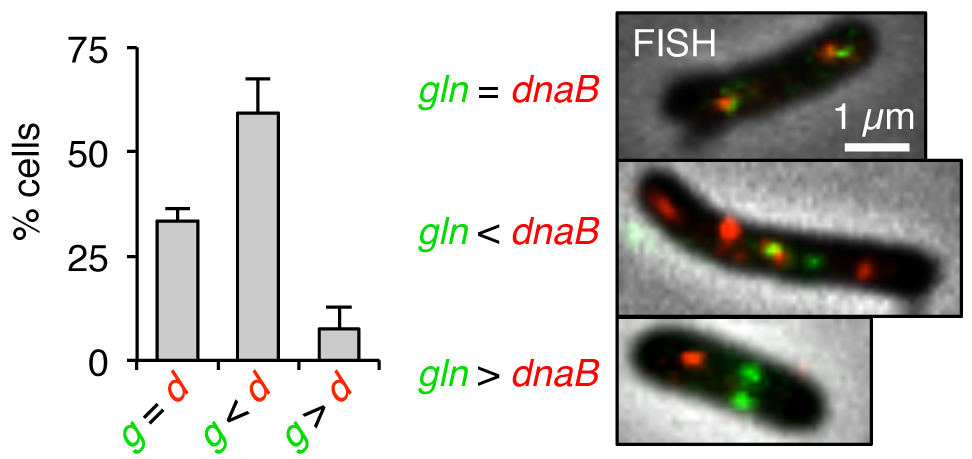

Supplement: Figure S5 — Two-color FISH analysis of gln and dnaB in parE10 cells. parE10 cells were grown to exponential phase in minimal succinate media at 30°C, shifted to 42°C for 2 hours, then analyzed by two-color FISH at gln and dnaB loci (Materials and Methods). The majority of cells contained fewer gln foci than dnaB foci, indicating greater cohesion at gln in the absence of Topo IV (±1 SD of 3 independent experiments, 300 cells each). (TIF) [file pgen.1003673.s005.tif]

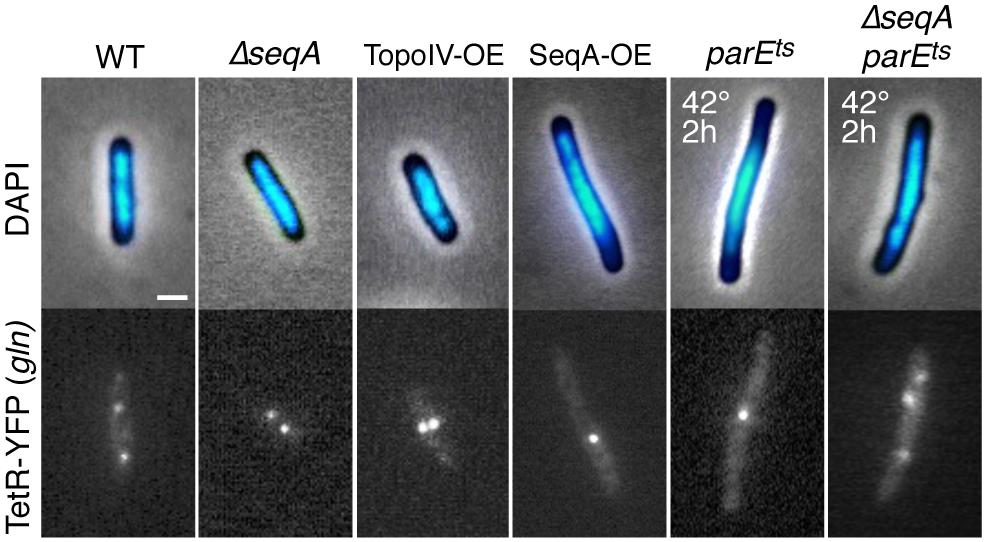

Supplement: Figure S6 — Nucleoid and gln-YFP micrographs in cells deficient in or overexpressing SeqA and Topo IV. Representative cell images from Figure 5C are shown. (TIF) [file pgen.1003673.s006.tif]

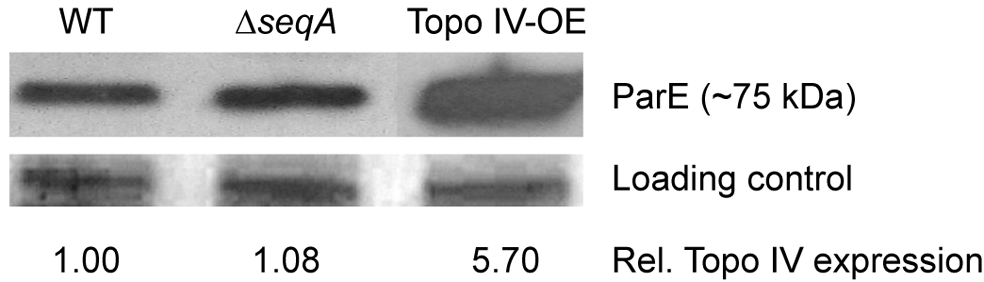

Supplement: Figure S7 — Topo IV expression analysis. Western blot analysis of Topo IV levels in WT, ΔseqA, and cells overexpressing Topo IV. Equal amounts of total protein (2 µg/ml) were loaded per lane. Blot was probed with monoclonal mouse anti-ParE (a kind gift of Lynn Zechiedrich) at 1∶10,000, detected with anti-mouse horseradish peroxidase, and imaged and quantified on a Storm Phosphorimager. Normalized intensities of ParE band relative to WT are shown. Topo IV overexpression was carried out using pDB332 as described in Figure 6. (TIF) [file pgen.1003673.s007.tif]

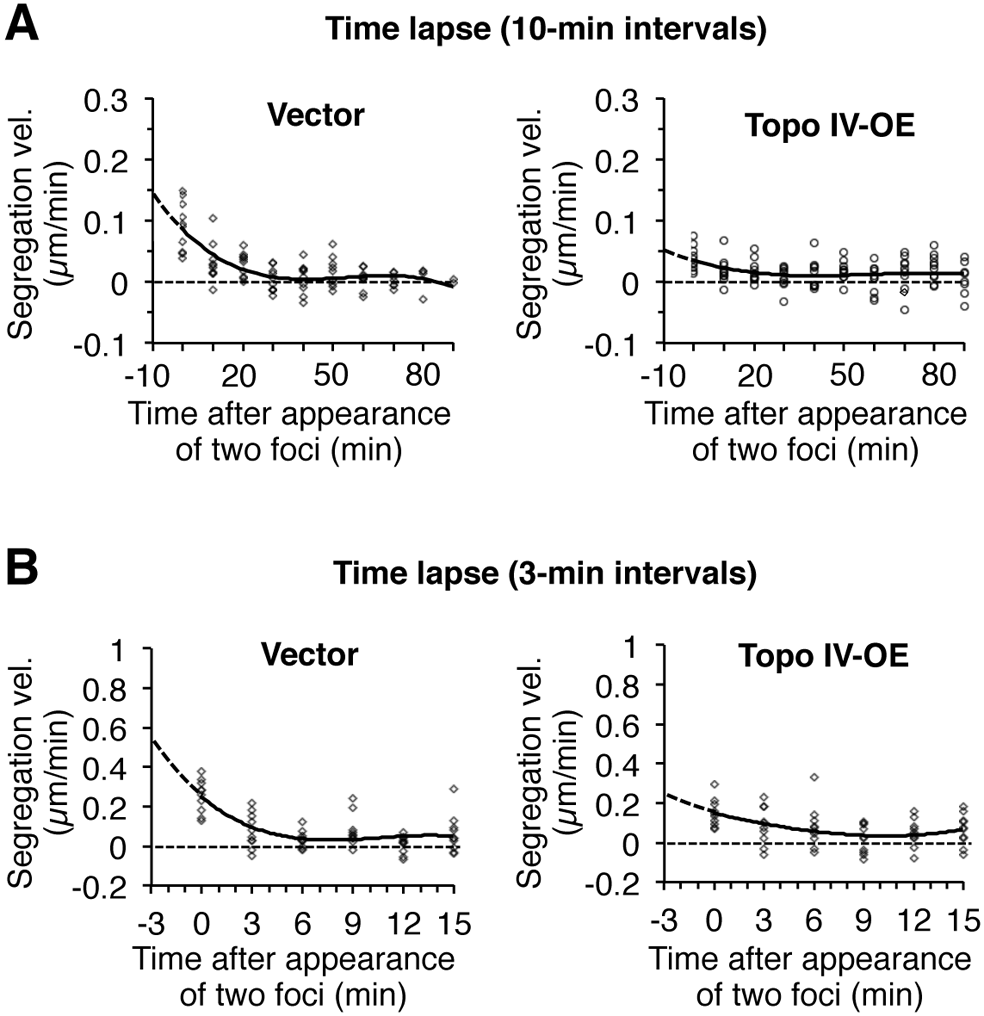

Supplement: Figure S8 — Segregation velocities in cells overexpressing Topo IV. Focus separation velocity was assessed at gln by FROS timelapse in Topo IV overexpressing (Topo IV-OE) or control (Vector) cells as described in Figure 6. (A) Change in rate of segregation of sister loci (net positive separation between consecutive time points) was plotted for the 10-minute interval movies shown in Figure 6A. (B) Three-minute interval movies were acquired through the first 15 minutes after focus splitting and sister separation was plotted as above. (TIF) [file pgen.1003673.s008.tif]

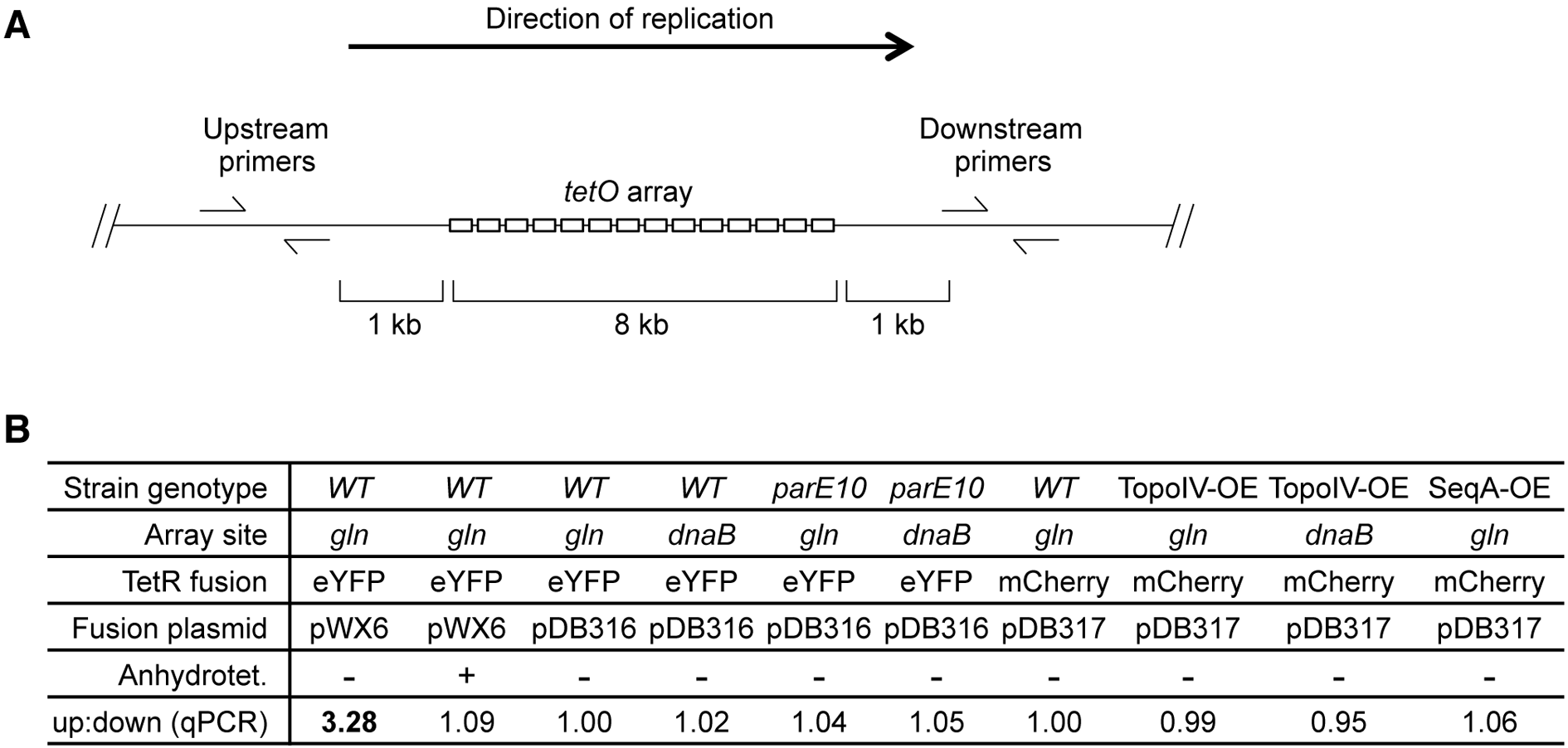

Supplement: Figure S9 — Current FROS system does not cause replication roadblocks. (A) Replication fork pausing at repressor-bound array sites was determined by qPCR analysis of segments immediately upstream and downstream of the array insertion. (B) Upstream∶downstream ratios ≈1.0 in all FROS strains used in the current study indicate an absence of replication pausing at the array site. For comparison, cells expressing TetR-YFP from pWX6 [54] in the absence of anhydrotetracycline (left-most column) have a >3-fold increase in DNA upstream of the array, indicating significant fork blockage. (TIF) [file pgen.1003673.s009.tif]

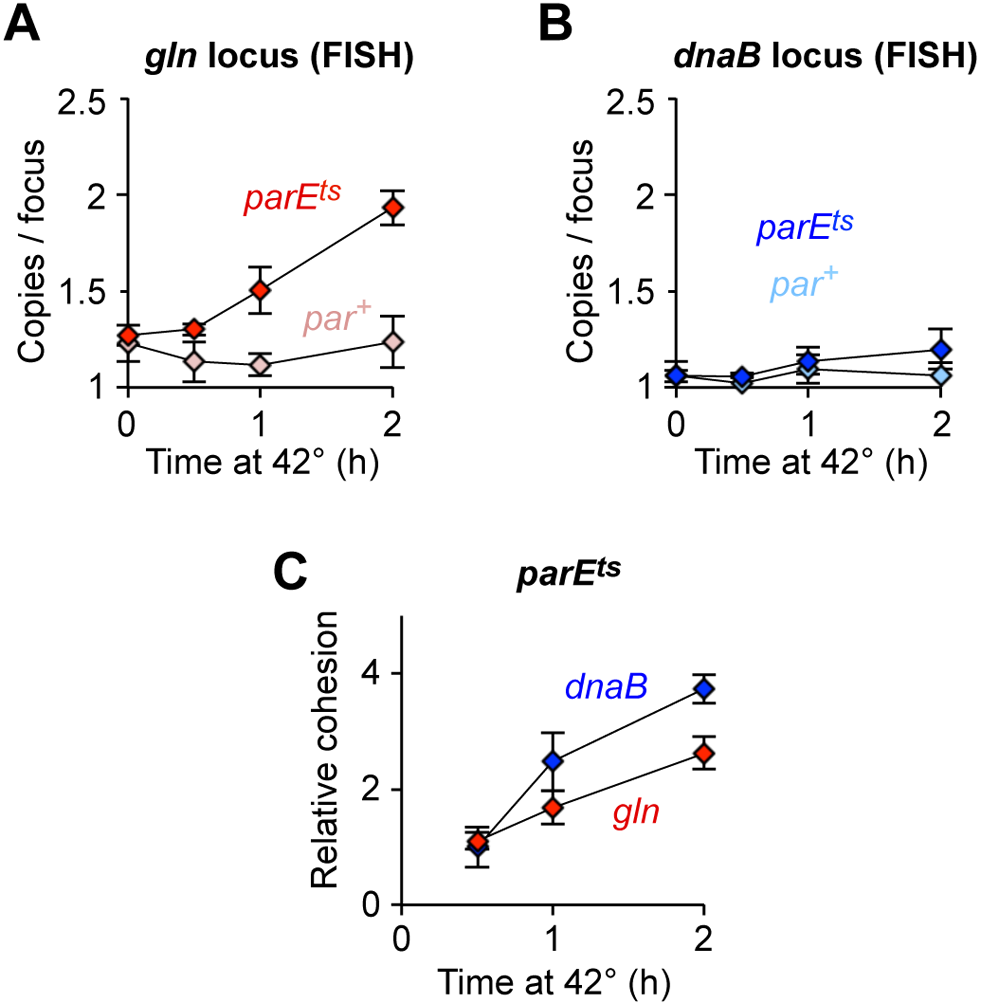

Supplement: Figure S10 — Confirmation of FROS cohesion timing results with FISH. Wild-type and parE10 cells without a tetO array were grown and analyzed as described in Figure 4, except that foci (segregation) were assayed by FISH (Materials and Methods). Results indicate that high cohesion in the absence of Topo IV is not an artifact TetR-YFP bound tetO arrays at the locus of interest. (A–B) Copy number per gln FISH focus (A) and per dnaB FISH focus (B) in parE10 (dark shaded symbols) and par+ control (light shaded symbols) cells after shift to restrictive temperature. Values are means of 3 experiments ±1 SD. (C) Relative cohesion at gln and dnaB in parE10 cells. (TIF) [file pgen.1003673.s010.tif]

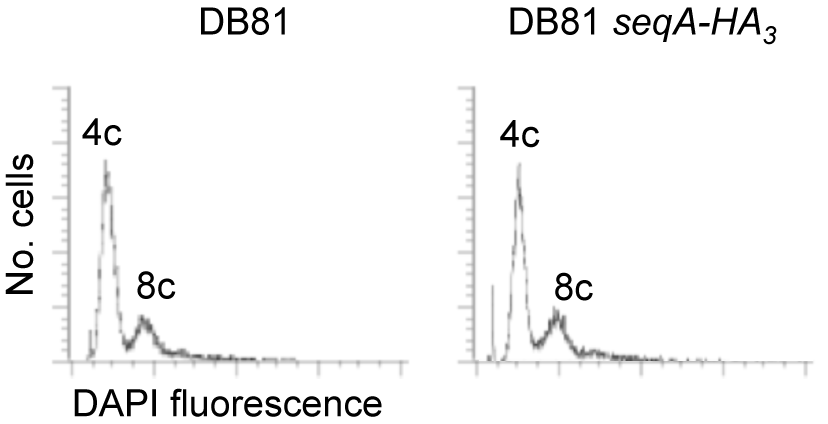

Supplement: Figure S11 — SeqA-HA3 protein provides normal replication initiation. WT (DB81) and seqA-HA3 strains were grown in LB at 37°C and assayed for origins per cell by Rifampicin runoff as in Figure S1. Synchronous replication initiation as shown by 2n origins, indicates that HA-tagged SeqA is fully functional. (TIF) [file pgen.1003673.s011.tif]
