# Supplementary material for: Regulation of Sister Chromosome Cohesion by the Replication Fork Tracking Protein SeqA
Source: PLoS Genet. 2013 Aug 22;9(8):e1003673. doi: 10.1371/journal.pgen.1003673 (PMC3749930; doi:10.1371/journal.pgen.1003673)
Supplement: Table S1 — Lists sequences for all cloning, qPCR and FISH probe primers. (PDF) [file pgen.1003673.s014.pdf]

**SUPPORTING INFORMATION**

**Table S1. PCR primers**

| | |
|---|---|
| **Topo IV expression plasmid cloning** | |
| *parC* (Forward) | 5’-CCGGAATTCATGAGCGATATGGCAGAGCGCCTTGCG-3’ |
| *parC* (Reverse) | 5’-AGGGGTACCTTACTCTTCGCTATCACCGCTGCTGGC-3’ |
| *parE* (Forward) | 5’-CCGGAATTCATGACGCAAACTTATAACGCTGATGCC-3’ |
| *parE* (Reverse) | 5’-CCCAAGCTTTTAAACCTCAATCTCCGCCATGTC-3’ |
| **qPCR** | |
| *oriC* (Forward) | 5’-TTCGATCACCCCTGCGTACA-3’ |
| *oriC* (Reverse) | 5’-CGCAACAGCATGGCGATAAC-3’ |
| *gln* (Forward) | 5’-TCAGTTTCGCTGGGAAATGTT-3’ |
| *gln* (Reverse) | 5’-GAATGGACGCTTCATTGTCGG-3’ |
| *dnaB* (Forward) | 5’-ACCGGAAGAAGGTTTACGGAT-3’ |
| *dnaB* (Reverse) | 5’-ATCCGGGATCTGGATTTCATG-3’ |
| *ter* (Forward) | 5’-AATGATGCCGGTTACCCAAAG-3’ |
| *ter* (Reverse) | 5’-AGTTGCGTTTCGACGGTCATT-3’ |
| **FISH probes** | |
| *dnaB* (Forward) | 5’-TTCAACAAACAGCAGGCTGAACCC-3’ |
| *dnaB* (Reverse) | 5’-ATTGGGCCGTTACGTTGTTTACCG-3’ |
| *gln* (Forward) | 5’-AATTGCTTCGTCAGTGAACACGCC-3’ |
| *gln* (Reverse) | 5’-ATCCCTGCTCATCAGGTGAATGCT-3’ |
